# Factors affecting the effectiveness and safety of colistin in treating drug-resistant gram-negative bacterial infections: a meta-analysis

**DOI:** 10.3389/fphar.2025.1625595

**Published:** 2025-10-29

**Authors:** Pengfei Li, Shiran Li, Jiao Zhang, Siyu Zeng, Zhimin Li, Jinxian Xie, Yong Yang

**Affiliations:** ^1^ Department of Pharmacy, Personalized Drug Research and Therapy Key Laboratory of Sichuan Province, Sichuan Provincial People’s Hospital, School of Medicine, University of Electronic Science and Technology of China, Chengdu, China; ^2^ Department of Pharmacy, Bishan hospital of Chongqing medical university, Bishan Hospital of Chongqing, Chongqing, China

**Keywords:** colistin, drug-resistant gram-negative bacteria, mortality, AKI, influencing factors

## Abstract

**Purpose:**

As an important antibiotic for treating infections caused by drug-resistant Gram-negative bacteria, colistin’s clinical efficacy and safety might be influenced by multiple factors. This meta-analysis aimed to identify the key factors affecting the effectiveness and safety of colistin in treating these infections. The results of this study provide a reference for clinicians to choose treatment methods. At the same time, the rational use of colistin can prevent the occurrence of adverse drug reactions (AKI), improve the cure rate of patients, and delay the development of bacterial resistance.

**Methods:**

The overall mortality rate was designated as the primary effectiveness outcome, with clinical response rate and bacterial eradication rate serving as the secondary outcomes. The incidence of acute kidney injury (AKI) was evaluated as a safety endpoint. Key analytical variables included colistin dose (high-dose≥4.2 mg/kg/day and low-dose< 4.2 mg/kg/day), ACCI (low <5, moderate = five to six, high >6), co-therapy (carbapenems/tigecycline/fosfomycin, etc.), microbial species (*Acinetobacter baumannii/Pseudomonas aeruginosa/*Enterobacteriaceae, etc.), and administration methods (aerosolized plus intravenous colistin vs. intravenous colistin alone).

**Results:**

A total of 74 studies (N = 8,889 participants) were included in our analysis. Mortality was lower in the high-dose group compared to the low-dose group (34.09% vs. 41.08%, p = 0.09). In ACCI score subgroups (low, moderate, high), mortality rates were 27.11% vs. 44.69% vs. 47.11% (p < 0.01). Monotherapy was associated with a higher mortality rate compared to co-therapy (42.97% vs. 33.10%, p < 0.01). Although no statistical differences were observed among different pathogenic bacteria species, infection caused by *A*. *baumannii* exhibited the highest mortality rate at 43.75%. Mortality rates for aerosolized plus intravenous colistin *versus* intravenous colistin alone were 40.81% vs. 32.84% (p = 0.09). The incidence of AKI was significantly higher in the loading dose group, high-ACCI group, and group receiving concomitant nephrotoxic drugs while being notably lower in the *Pseudomonas aeruginosa* infection group.

**Conclusion:**

Loading dose, co-therapy (carbapenems or quinolones), microbial factors, and ACCI are the main factors associated with the effectiveness of colistin. Additionally, loading dose, microbial factors, ACCI, and co-therapy are associated with an increased risk of colistin-associated AKI.

**Systematic Review Registration:**

https://www.crd.york.ac.uk/PROSPERO/search, identifier CRD420250655507.

## 1 Introduction

Drug-resistant Gram-negative bacterial (DR-GNB) infections, particularly those caused by carbapenem-resistant organisms (CRO), currently pose one of the most serious challenges to global public health (Holmes et al., 2021). It is crucial to note that infections caused by CRO, such as pneumonia, sepsis, and complicated intra-abdominal infections, are associated with high prevalence and significant mortality rates (Gurjar et al., 2013; Shah and Shah, 2015). This global crisis is exacerbated by the fact that CROs exhibit resistance to the majority of currently available antibiotics, severely limiting therapeutic options. In this context, colistin has emerged as the last-line therapeutic option for combating CRO infections (Lu et al., 2022). However, the clinical utility of colistin is now being challenged by the emergence and global dissemination of plasmid-mediated colistin resistance genes (*mcr*). These genes have been identified in isolates from both animal and environmental sources across multiple countries, and clinical isolates harboring *mcr* genes are being increasingly reported (Mohsin et al., 2018; Papst et al., 2018). To date, more than 11 *mcr* gene subtypes have been identified (Li et al., 2017), highlighting the urgent need to strengthen prevention and control measures against polymyxin antimicrobial resistance.

Colistin is a concentration-dependent, cationic polypeptide antibiotic that lacks a post-antibiotic effect (Gunderson et al., 2003). It is administered intravenously in the form of colistin methanesulfonate sodium (CMS), which exerts its antibacterial activity by binding to bacterial lipopolysaccharide and disrupting the integrity of the outer membrane. CMS is primarily eliminated through renal excretion and may undergo renal tubular reabsorption (Li et al., 2004; Kristoffersson et al., 2020). The clinical effectiveness of colistin is believed to be influenced by a variety of factors, including dosing strategy, co-therapy with other antimicrobial agents, adjunctive aerosolized therapy, and baseline clinical characteristics, among others. However, existing studies have not demonstrated consistent findings (Nation et al., 2017; Ahmadpour et al., 2022). Moreover, due to its significant nephrotoxicity, specifically AKI, colistin was once regarded as a suboptimal choice. Whether colistin-associated AKI has clinical importance remains a matter of debate (Dalfino et al., 2012; Gurjar et al., 2013). While early clinical and preclinical studies reported significant rates of nephrotoxicity and neurotoxicity following systemic administration, more recent data suggest that colistin may be safer than previously thought, with a lower incidence of AKI in some cohorts (Falagas and Kasiakou, 2005). Prior studies indicate a 20%–60% incidence of colistin-associated AKI, which is likely influenced by a multitude of factors including baseline glomerular filtration rate, severity of infection, hemodynamic status, colistin dose, and concomitant use of nephrotoxic drugs. In addition, some studies have shown that colistin-associated AKI occurs early, is not severe, and does not lead to discontinuation of colistin (Gurjar et al., 2013).

To address these controversies, this meta-analysis aims to comprehensively analyze the factors influencing the effectiveness and safety of colistin in treating drug-resistant Gram-negative bacterial infections. We take into account three aspects: drugs, microorganisms, and patient-related factors. From the drug perspective, we focus on the daily dose, combination drug therapy, and adjuvant aerosolized administration; and for microorganisms, emphasis is placed on pathogenic bacteria species, for patient-related factors; key considerations include age and comorbidities.

## 2 Materials and methods

Our meta-analysis was conducted in strict accordance with the PRISMA 2020 guidelines (Rethlefsen and Page, 2021), and has been registered in PROSPERO (Registration No.: CRD420250655507).

### 2.1 Data sources and search strategy

A comprehensive search was conducted in PubMed, Embase, the Cochrane Library, and Web of Science for studies published between 1 January 2000, and 31 December 2024. The search strategy incorporated combinations of the following keywords: colistin, colistin methanesulfonate sodium (CMS), drug-resistant bacteria, multidrug-resistant Gram-negative bacteria (MDR), or carbapenem-resistant organisms (CRO), to construct the search strategy. Additionally, to retrieve relevant studies as comprehensively as possible, reference lists of eligible articles including randomized controlled trials (RCTs), cohort studies, case-control studies, systematic reviews, and meta-analyses were also manually screened. Only English-language articles involving human subjects were included. To enhance reproducibility, the full search strategies for all databases are provided in Supplementary Material 1 (including PubMed, Embase, the Cochrane Library, and Web of Science).

### 2.2 Inclusion and exclusion criteria

Inclusion and exclusion criteria were strictly defined based on the PICOS framework.

#### 2.2.1 Inclusion criteria

Studies were included if they met the following conditions: ① Studies involving human participants. ② The infection was microbiologically confirmed to be caused by resistant Gram-negative bacteria (including drug-resistant *Pseudomonas aeruginosa, Acinetobacter baumannii*, and Enterobacteriaceae). ③ Colistin was used either as monotherapy or in combination therapy. ④ At least one outcome of interest was available, including mortality, bacterial clearance, clinical response, or AKI incidence. ⑤ Colistin was administered for a minimum of 3 days ⑥ Study design was a randomized controlled trial or observational study.

#### 2.2.2 Exclusion criteria

Studies were excluded if they met any of the following: ① Studies conducted *in vitro* or on animals. ② Enteral or ophthalmic administration of colistin. ③ Studies using colistin sulfate for injection were used. ④ Articles not classified as research papers (e.g., reviews, editorials). ⑤ Studies not published in English.

### 2.3 Study selection

First, two of the authors (LP and LS) independently conducted the initial screening of all article titles and abstracts based on the predefined search strategy and inclusion/exclusion criteria, selecting studies that met the eligibility requirements based on their respective judgments. Subsequently, the two authors independently re-examined the full texts of their initially selected studies to assess eligibility criteria, ultimately determining their respective final inclusions through this secondary evaluation process. Finally, the two authors jointly compared and analyzed the studies they each ultimately included. Any discrepancies were resolved through discussion to reach a consensus. When consensus could not be achieved, a third author (YY) was consulted to adjudicate the disagreements.

### 2.4 Outcomes selection

Overall mortality rate is widely accepted as a key indicator of patient outcomes. The main adverse reaction of colistin is nephrotoxicity, manifested as elevated creatinine levels, decreased urine output, and AKI, with AKI being the most severe. Therefore, we defined overall mortality (including but not limited to 7-day, 14-day, 28-day, and 30-day mortality rates) as the primary efficacy outcome of our meta-analysis. The secondary outcomes included clinical response rate and bacterial eradication rate, while AKI was designated as the primary safety outcome. For the criteria of AKI, we adopt the KDIGO criteria to define nephrotoxicity, which includes meeting one of the following conditions: (1) Serum creatinine (Scr) increases by more than 26.5 μmol/L (0.3 mg/dL) within 48 h; (2) Scr rises to more than 1.5 times the baseline level within 7 days; (3) Urine output <0.5 mL/(kg·h), persisting for more than 6 h (de Boer et al., 2022). The original study included in the AKI study provided data based on at least one of the 3 KDIGO criteria for all patients.

### 2.5 Data extraction and processing

Data extraction was performed independently by two authors using a standardized electronic spreadsheet. Disagreements were resolved through consensus. The extracted data included: study characteristics (first author, study location, study type, sample size, etc.), patient characteristics (patient age, gender, weight, infection site, microbial type, comorbidities), treatment methods (daily dose of colistin, course of treatment, administration method, concomitant medication, etc.), and final outcomes (mortality, bacterial clearance rate, clinical efficacy, and AKI incidence, etc.). RevMan was used for data management and analysis. To address missing data, we attempted to query ClinicalTrials.gov and contacted the authors of the included studies whenever possible to obtain more comprehensive information.

In these studies, colistin was administered in the form of its prodrug CMS. Due to differences in units of measurement between countries, we performed dose conversion and expressed the results using colistin base activity to facilitate statistical analysis. Numerous previous studies have demonstrated that the steady-state plasma concentration (Css) serves as the most direct determinant of a drug’s antimicrobial efficacy. When the total daily dose remains constant, body weight significantly influences plasma drug concentrations. To minimize the impact of body weight on Css, we tried to use the dose conversion method to obtain an indicator that can more accurately reflect the drug plasma concentration - unit weight dose. We defined the unit weight dose of the drug as the daily dose/average weight of the study population. We used the standard maintenance dose recommended in the latest international consensus guidelines - 300 mg CBA (∼9 million IU CMS) divided into two and infused at 12-h intervals - as the critical value of the daily dose (Tsuji et al., 2019). Using the ratio of this critical value to the average weight of each study population (71.4 kg), we finally obtained a critical value of 4.2 mg/kg/day CBA for the dose per kilogram of body weight. Additionally, when sample weight data were unavailable, we opted to substitute it with the average of available adult weight data within the sample, thereby minimizing the impact of weight discrepancies on plasma drug concentration.

We used comorbidities and age to measure the impact of baseline pathological status and physiological status on treatment, respectively. In two recently published large-scale, multicenter studies on the death risk assessment model for sepsis patients based on ACCI, the ACCI risk was divided into three levels: low risk (<5), moderate risk (5–6), and high risk (>7) (Liu et al., 2025; Cheng et al., 2025). They concluded that there were significant differences between the three subgroups. Therefore, we also referred to their conclusions for the risk level division of ACCI. In cases where the original study did not provide an ACCI score, but did provide data on patient comorbidities, we calculated the mean ACCI based on the scoring criteria described above.

### 2.6 Quality assessment

The two authors responsible for data extraction independently conducted risk-of-bias assessments. The Newcastle-Ottawa Scale (NOS) was used for observational studies and the Cochrane Risk-of-Bias Tool for RCTs. If scoring discrepancies arise, they shall be resolved through panel discussion or adjudication by a third reviewer. Only RCTs and high-quality observational studies (NOS ≥7) were included in the final analysis. All statistical analysis was performed using R 4.2.3. The prevalence with associated 95% confidence intervals was used to assess outcomes.

### 2.7 Heterogeneity

Given the anticipated variability in study design and population characteristics, heterogeneity was assessed using forest plots under a random-effects model, eliminating the influence of the number of studies on the statistical power of hypothesis testing, and utilized I^2^ for quantitative assessment. To investigate potential sources of heterogeneity, we performed a multivariate meta-regression analysis, incorporating study type (RCT vs. observational study), patient age, continent, and treatment duration as covariates. This model as a whole was able to significantly explain the heterogeneity between studies. Post-hoc sensitivity analyses were also performed to test the robustness of pooled results.

### 2.8 Publication bias

Publication bias was evaluated using Egger’s test for the included studies.

## 3 Results

### 3.1 Search results

**FIGURE 1:**
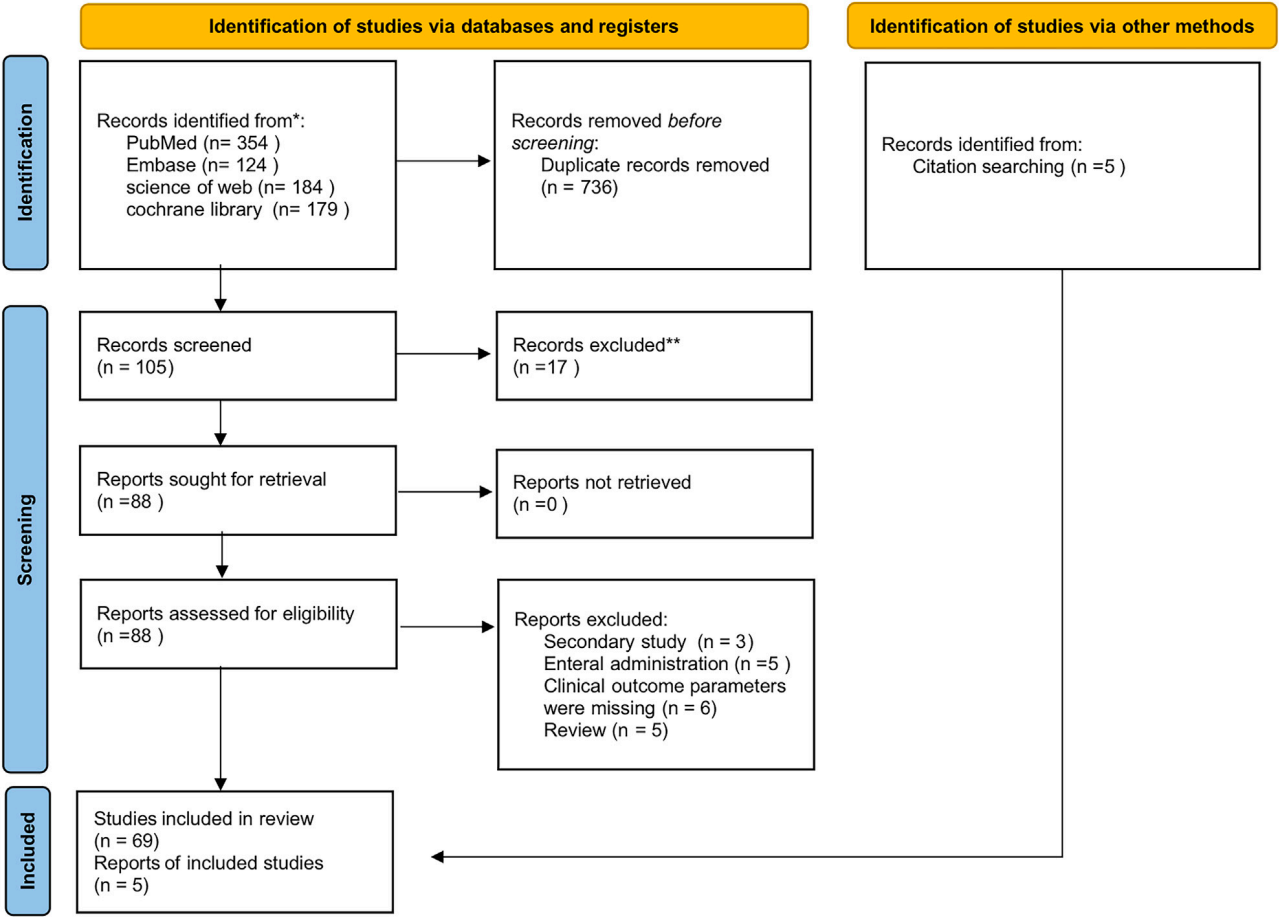
PRISMA flow diagram of study inclusion and exclusion.

Through predefined search strategies across four target databases, a total of 841 potentially relevant studies were initially identified. During the deduplication process, 457 duplicate studies were removed. Subsequently, title and abstract screening led to the exclusion of 105 studies. Ultimately, a full-text review and detailed analysis yielded a final selection of 69 studies for inclusion. Additionally, five more studies were identified by screening the reference lists of relevant systematic reviews and meta-analyses. A detailed flowchart of the search strategy is presented in Figure 1.

### 3.2 Study characteristics

The included studies comprised 33 RCTs, 33 retrospective cohort studies, six prospective observational studies, and 2 case-control studies. Among these, mortality was reported in 62 studies, clinical response rate in 57 studies, bacterial eradication rate in 43 studies, and incidence of AKI in 51 studies. A total of 8,889 patients were enrolled in our study, of whom 8,038 received colistin treatment. Table 1 summarizes the relevant information on the ultimately included studies. Detailed information of the finally selected studies can be found in Supplementary Material 2.

**TABLE 1:** Summary of included studies.

Variable	n (%)
Study number	74
Patients number	8,038
Male	4,717 (58.68%)
Female	3,321 (41.32%)
Study type	
RCT	33 (44.59%)
Retrospective cohort study	33 (44.59%)
Prospective observational study	6 (8.11%)
Case-control study	2 (2.71%)
Dose^a^	
High dose, ≥4.2 mg/kg/d	4,646 (57.8%)
Low dose, <4.2 mg/kg/d	1,789 (22.3%)
Antibiotic-resistant bacterial species^b^	
CRPA	639 (7.96%)
CRAB	5,672 (70.56%)
CRKP	534 (6.63%)
CRE	408 (5.07%)
Mixed or other Gram negatives bacteria	785 (9.77%)
Site of infection	
Pneumonia (HAP/VAP)	5,218 (64.92%)
Urinary tract infections (UTI)	268 (3.34%)
Bloodstream Infection (BSI)	1,414 (17.58%)
Multisite Infection	1,138 (14.16%)

^a^ Missing weight data were imputed using the mean weight of the available sample data.

^b^ CRAB, carbapenem-resistant *A. baumannii*; CRPA, carbapenem-resistant *Pseudomonas aeruginosa*; CRPK, carbapenem-resistant *K. pneumoniae*; CRE, carbapenem-resistant Enterobacterales.

Table 2 summarizes the impact of each influencing factor on various outcome measures associated with colistin treatment for infections caused by drug-resistant Gram-negative bacteria.

**TABLE 2:** The impact of each influencing factor on various outcome measures.

		Efficacy outcome						Safety outcome	
		Overall mortality (%)		Clinical response rate (%)		Bacterial eradication rate (%)		AKI (%)	
Does	high-does	34.09	p = 0.09	60.65	p = 0.40	58.73	p = 0.65	28.55	p = 0.74
	low-does	41.08		56.45		61.16		26.92	
	loading dose	33.33	**p < 0.01**	NA	NA	NA	NA	31.83	**p = 0.03**
	non-loading dose	49.16		NA		NA		19.97	
ACCI	low-ACCI	27.11	**p < 0.01**	74.40	**p < 0.01**	64.82	P = 0.97	13.71	**p < 0.01**
	moderate-ACCI	44.69		64.35		63.63		29.43	
	high-ACCI	47.11		54.11		63.81		31.98	
Co-therapy	monotherapy	42.97	**p < 0.01**	42.04	**p < 0.01**	60.02	**p < 0.01**	20.75	**p < 0.01**
	carbapenems	30.57		67.02		47.29		27.25^a^	
	tigecycline	50.64		NA		NA		50.88**	
	rifampicin	46.17		75.69		65.03			
	quinolones	23.87		86.63		63.82			
	fosfomycin	30.12		NA		98.17			
	mixed antibiotics	33.10		57.73		60.35		16.08	
Microbial species	CRAB	43.75	**p < 0.01**	58.83	**p = 0.05**	59.31	**p < 0.01**	28.02	**p < 0.01**
	CRPA	24.40		72.97		45.05		12.27	
	CRE	28.69		61.47		81.07		21.30	
	CRKP	33.18		71.55		NA		28.22	
	mixed bacteria	31.29		57.68		57.47		25.89	
Administration methods	ivgtt	40.81	p = 0.09	57.50	p = 0.62	54.30	p = 0.69	30.43	p = 0.19
	ivgtt + ae	32.84		64.66		59.56		29.67	
	ae	22.38		37.20		60.56		16.88	

^a^ Combination Therapy Group I (carbapenems/tigecycline/β-lactams/quinolones), **Combination Therapy Group II (glycopeptides/loop diuretics/aminoglycosides).

CRAB, carbapenem-resistant *A. baumannii*; CRPA, carbapenem-resistant *P. aeruginos*a; CRPK, carbapenem-resistant *K. pneumoniae*; CRE, carbapenem-resistant Enterobacterales ivgtt, intravenous guttae; ivgtt + ae, intravenous guttae plus aerosolized; ae, aerosolized. Bold values indicate a significant statistical difference between different subgroups.

### 3.3 Efficacy outcome

#### 3.3.1 Colistin daily dose

**FIGURE 2:**
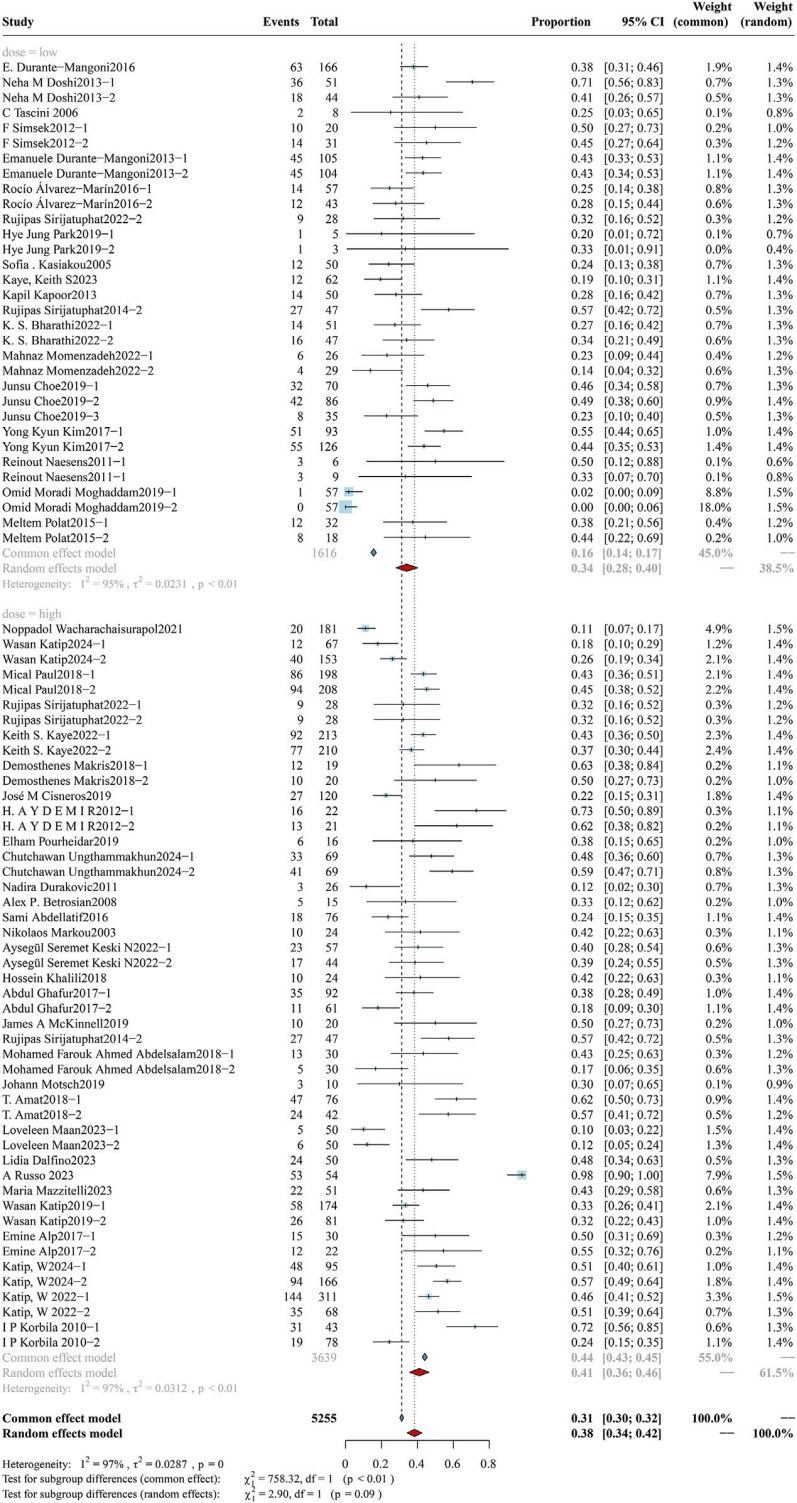
Forest plot of the effect of colistin daily dose (high vs. low) on overall mortality. The forest plot illustrates the impact of colistin daily dose on overall mortality in patients with drug-resistant Gram-negative bacterial infections. Studies were stratified into two subgroups based on dosing regimen: high-dose (≥4.2 mg/kg/day) and low-dose (<4.2 mg/kg/day).

**FIGURE 3:**
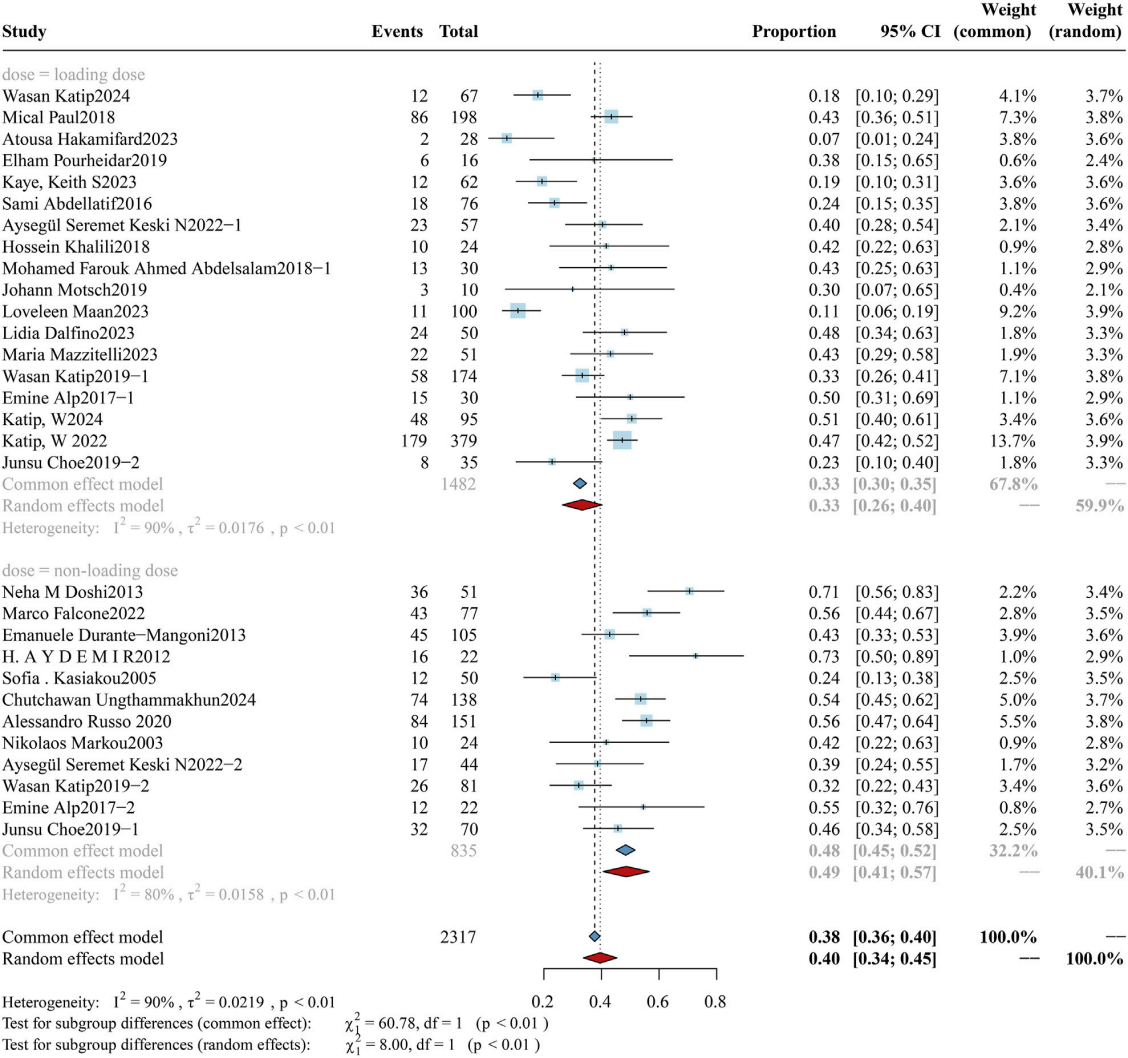
Forest plot of the effect of loading dose on overall mortality. The forest plot illustrates the impact of administering a colistin loading dose *versus* no loading dose on overall mortality in patients with drug-resistant Gram-negative bacterial infections. Subgroup analysis compares clinical outcomes between groups receiving a loading dose and those without.

According to the latest international consensus guidelines (Tsuji et al., 2019), the dosing regimen for colistin has been revised to a loading dose of 9 MIU, followed by an elevated maintenance dose of 4.5 MIU administered every 12 h. In this review, a daily dose of colistin was converted to X mg/kg/day according to the guidelines and subsequently stratified into high-dose and low-dose subgroups using a threshold of 4.2 mg/kg/day. The analysis of overall mortality in the two subgroups is shown in Figure 2, while clinical response and bacterial eradication rates are detailed in Supplementary Material 3 (Figures 1, 2). The daily dose study cohort comprised 6,435 participants, including 4,646 in the high-dose group and 1,789 in the low-dose group. Notably, the high-dose subgroup demonstrated a mortality rate of 34.09% (95%CI: 28.09%–40.08%), accompanied by a clinical response rate of 56.45% (95%CI: 50.23%–62.67%) and a bacterial eradication rate of 61.16% (95%CI: 53.17%–69.14%). In comparison, the low-dose subgroup reported a mortality rate of 41.08% (95%CI: 35.70%–46.45%), a clinical response rate of 60.65% (95%CI: 53.12%–68.19%), and a bacterial eradication rate of 58.73% (95%CI: 51.94%–65.51%). Variations in colistin dosing regimens (higher vs. lower daily doses) do not exhibit statistically significant differences in clinical outcomes (p > 0.05). A further subgroup analysis evaluated the impact of a loading dose on outcomes. The forest plot for overall mortality is presented in Figure 3. Notably, a statistically significant difference was observed between the two subgroups, with mortality rates of 33.33% (95%CI: 26.45%–40.21%) vs. 48.61% (95%CI: 40.56%–56.66%) (p < 0.01).

#### 3.3.2 ACCI

**FIGURE 4:**
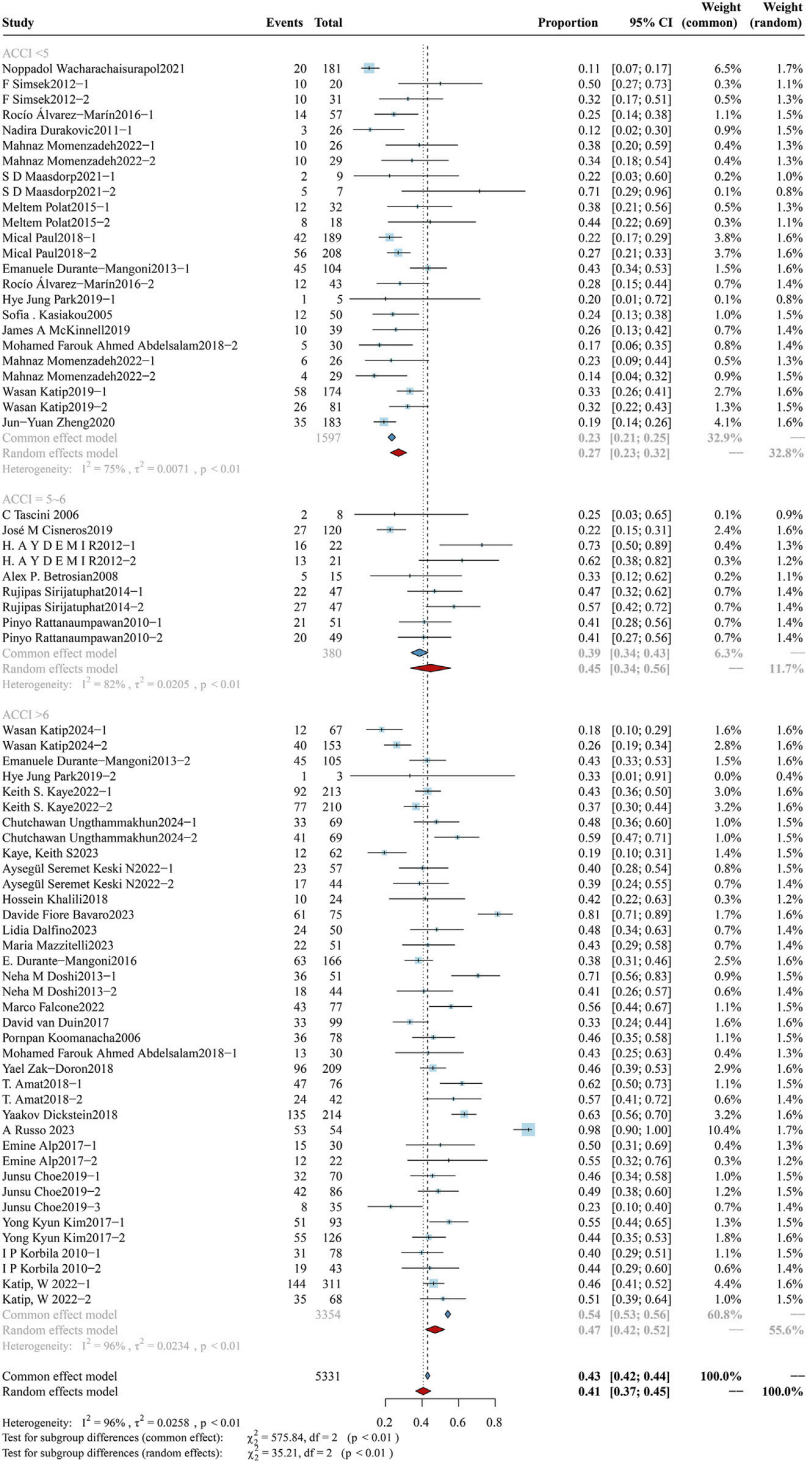
Forest plot of the effect of ACCI on overall mortality. The forest plot illustrates the impact of different age-adjusted Charlson comorbidity index (ACCI) levels on overall mortality among patients receiving colistin for drug-resistant Gram-negative bacterial infections. Subgroup analyses were conducted based on ACCI scores, categorized as <5, 5–6, and >6.

The ACCI serves as a comprehensive metric for evaluating patients’ overall baseline health status. Using this scoring system, participants were stratified into three groups: low-ACCI (<5), moderate-ACCI (5–6), and high-ACCI (>6). The mortality analysis of the three subgroups is shown in Figure 4, and the clinical response rate and bacterial eradication rate of these subgroups can be found in Supplementary Material 3 (Figures 3, 4). A total of 5,331 participants were recruited in the ACCI study cohort and divided into three subgroups: the low-ACCI subgroup included 1,597 participants (30.0%), the moderate-ACCI subgroup included 380 participants (7.1%), and the high-ACCI subgroup included 3,354 participants (62.9%). The mortality rate was significantly different among the subgroups: low-ACCI - 27.11% (95%CI: 22.70%–31.51%), moderate-ACCI subgroup - 44.69% (95%CI: 33.71%–55.66%), and high-ACCI subgroup was 47.11% (95%CI: 41.85%–52.37%) (p < 0.01). The clinical response rates also varied significantly between the groups: low-ACCI - 74.40% (95%: 67.25%–81.55%), moderate-ACCI subgroup - 64.35% (95%CI: 53.86%–74.85%), high-ACCI subgroup - 54.11% (95%CI: 47.35%–60.88) (p < 0.01). In contrast, bacterial eradication rates did not show significant differences between groups.

#### 3.3.3 Co-therapy

**FIGURE 5:**
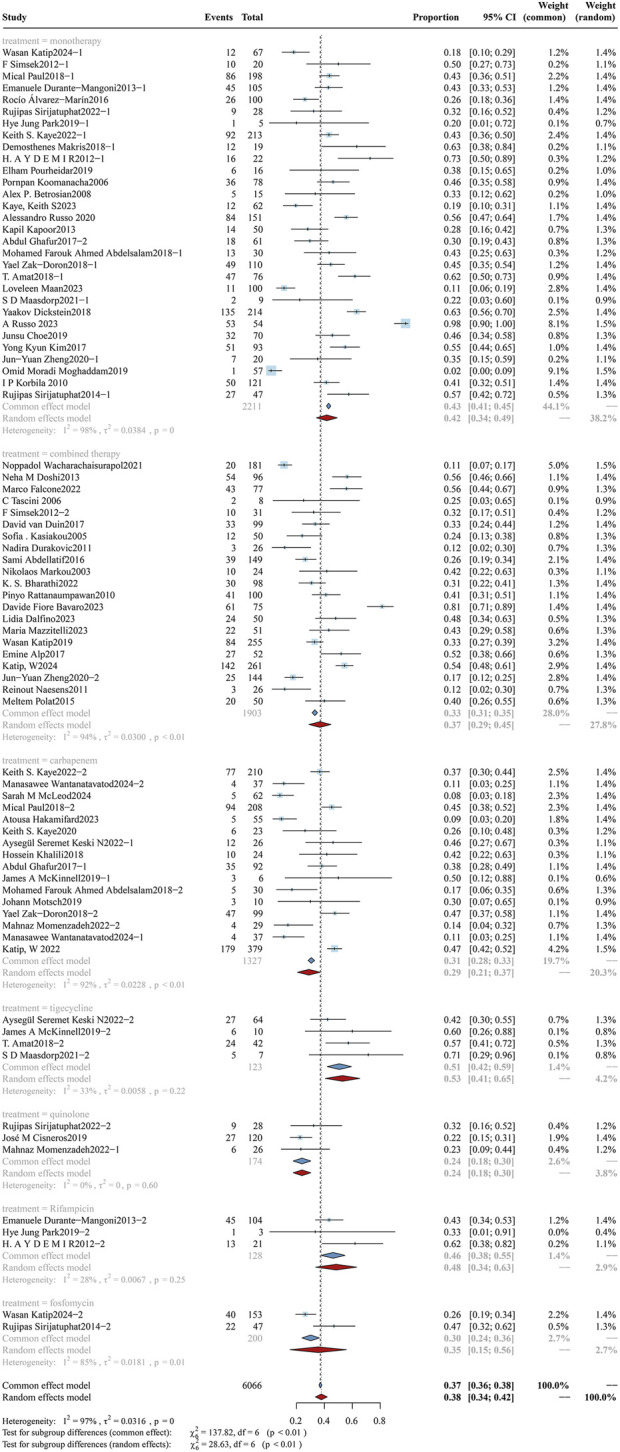
Forest plot of the effect of co-therapy on overall mortality. The forest plot presents the impact of different co-therapy regimens on overall mortality among patients treated with colistin for drug-resistant Gram-negative bacterial infections. Subgroup analyses were conducted based on the type of co-administered agent, including monotherapy, combined therapy (non-specified), carbapenem, tigecycline, quinolone, rifampicin, and fosfomycin.

**FIGURE 6:**
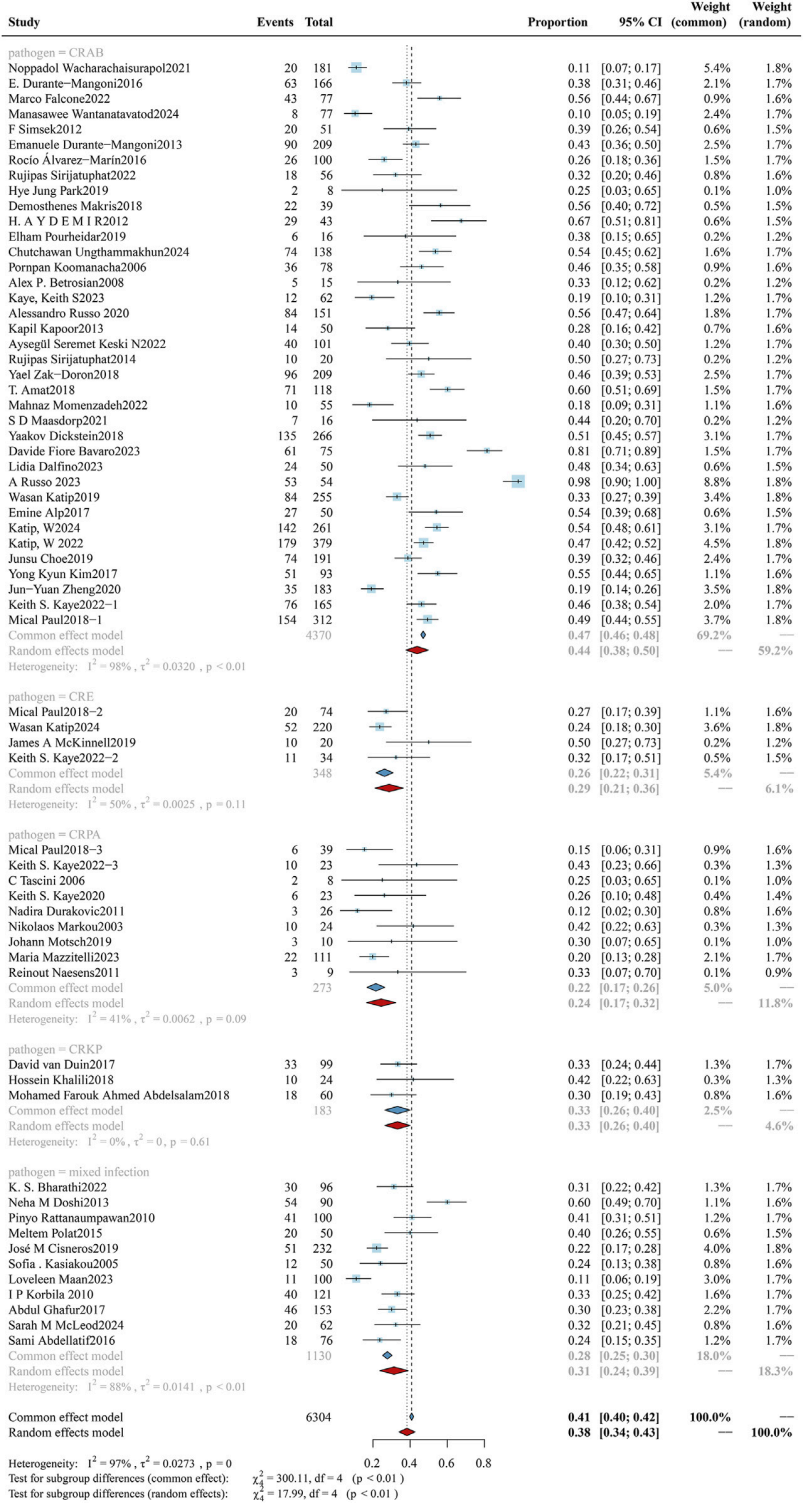
Forest plot of the effect of microbial species on overall mortality. The forest plot presents a subgroup analysis of overall mortality stratified by the causative Gram-negative pathogens in patients treated with colistin. Subgroups include carbapenem-resistant *A*. *baumannii* (CRAB), carbapenem-resistant *Enterobacterales* (CRE), carbapenem-resistant. *K*. *pneumoniae* (CRKP), carbapenem-resistant *Pseudomonas aeruginosa* (CRPA), and mixed infections involving multiple resistant Gram-negative species.

The cohort was stratified into subgroups according to clinically prevalent combination therapeutic strategies: monotherapy (colistin alone), tigecycline, rifampicin, quinolones, and β-lactams (carbapenems). Results of overall mortality analyses by subgroup are shown in Figure 5, with clinical efficacy and bacterial eradication rates displayed in Supplementary Material 3 (Figures 5, 6). The study cohort comprised a total of 7,676 patients: monotherapy group (n = 3,711), carbapenem group (n = 1,327), combination therapy group (n = 1,903), quinolone group (n = 174), tigecycline group (n = 123), rifampicin group (n = 238), and fosfomycin group (n = 200). The overall mortality rate in the monotherapy subgroup was 42.97% (95%CI: 41.43%–44.52%), with a clinical response rate of 42.04% (95%CI: 32.11%–51.98%) and a bacterial clearance rate of 60.02% (95%CI: 58.17%–61.87%). In contrast, the carbapenem subgroup had a lower mortality rate of 30.57% (95%CI: 28.26%–32.89%), an improved clinical response rate of 59.73% (95%CI: 58.68%–75.37%), and a higher bacterial clearance rate of 69.32% (95%CI: 67.19%–71.45%). The quinolone subgroup had an overall mortality rate of 23.87% (95%CI: 17.56%30.19%), a clinical response rate of 86.63% (95%CI: 73.38%–99.89%), and a bacterial clearance rate of 63.82% (95%CI: 51.22%–76.43%). In the rifampicin subgroup, the overall mortality rate was 46.17% (95%CI: 37.62%–54.71%), with a clinical response rate of 75.69% (95%CI: 42.38%–100.00%) and a bacterial eradication rate of 65.03% (95%CI: 56.85%–73.20%). In the antibiotic combination subgroup, the overall mortality rate was 33.10% (95%CI: 31.16%–35.04%), accompanied by a clinical response rate of 59.73% (95%CI: 52.55%–66.91%) and a bacterial eradication rate of 69.32% (95%CI: 67.19%–71.45%). Our findings demonstrate that the combination therapy of colistin with carbapenems and quinolones is associated with a statistically significant reduction in overall mortality (p < 0.01). Moreover, the combination of colistin and carbapenems significantly enhanced the clinical response rate (p < 0.01). The synergistic effect of drug combinations resulted in superior therapeutic outcomes relative to single-agent treatment.

#### 3.3.4 Microbial species

**FIGURE 7:**
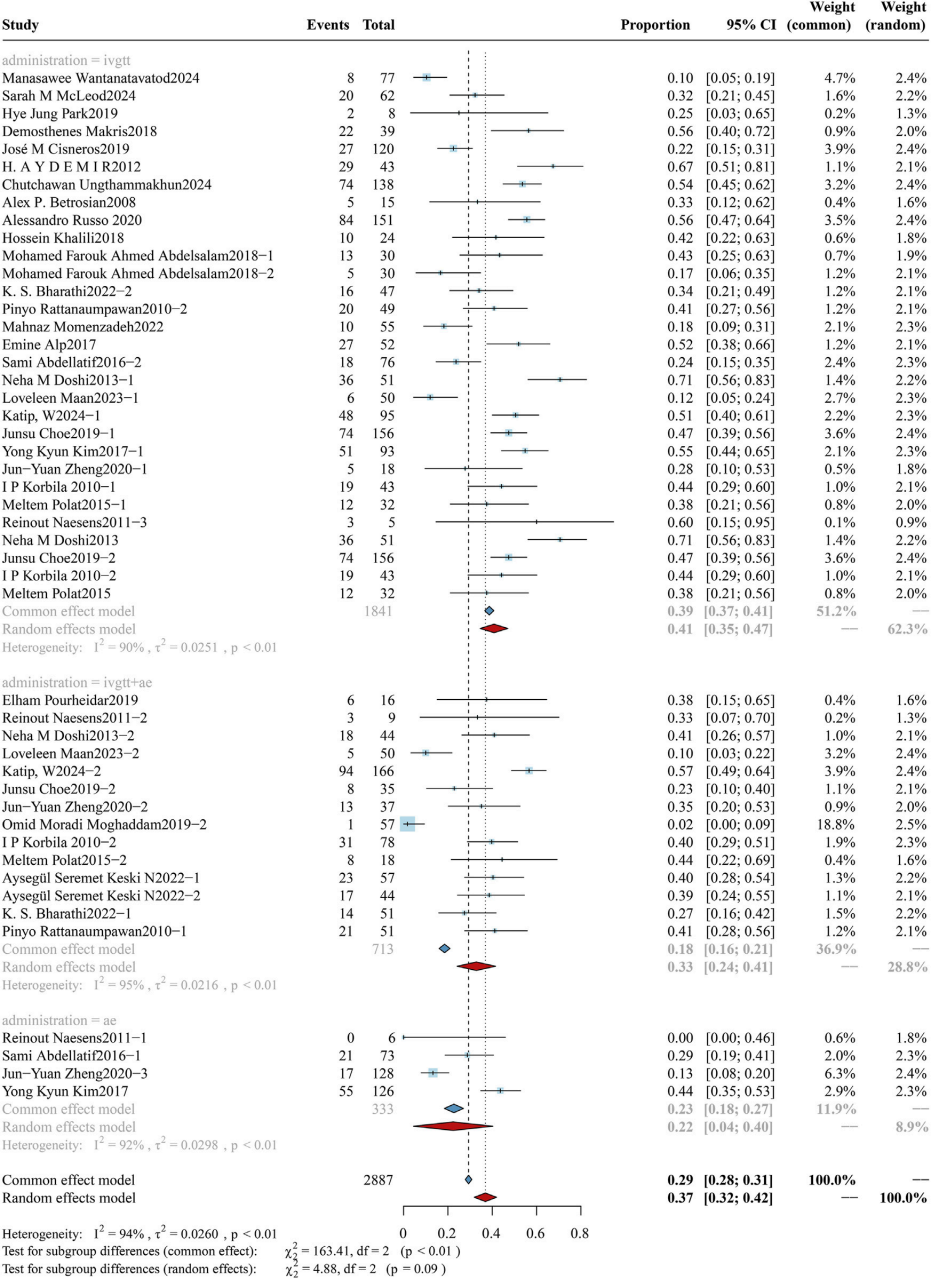
Forest plot of the effect of administration methods on overall mortality. The figure presents a subgroup analysis comparing the overall mortality associated with colistin treatment across three administration methods: intravenous monotherapy (ivgtt), combined intravenous and aerosolized therapy (ivgtt + ae), and aerosolized monotherapy (ae).

**FIGURE 8:**
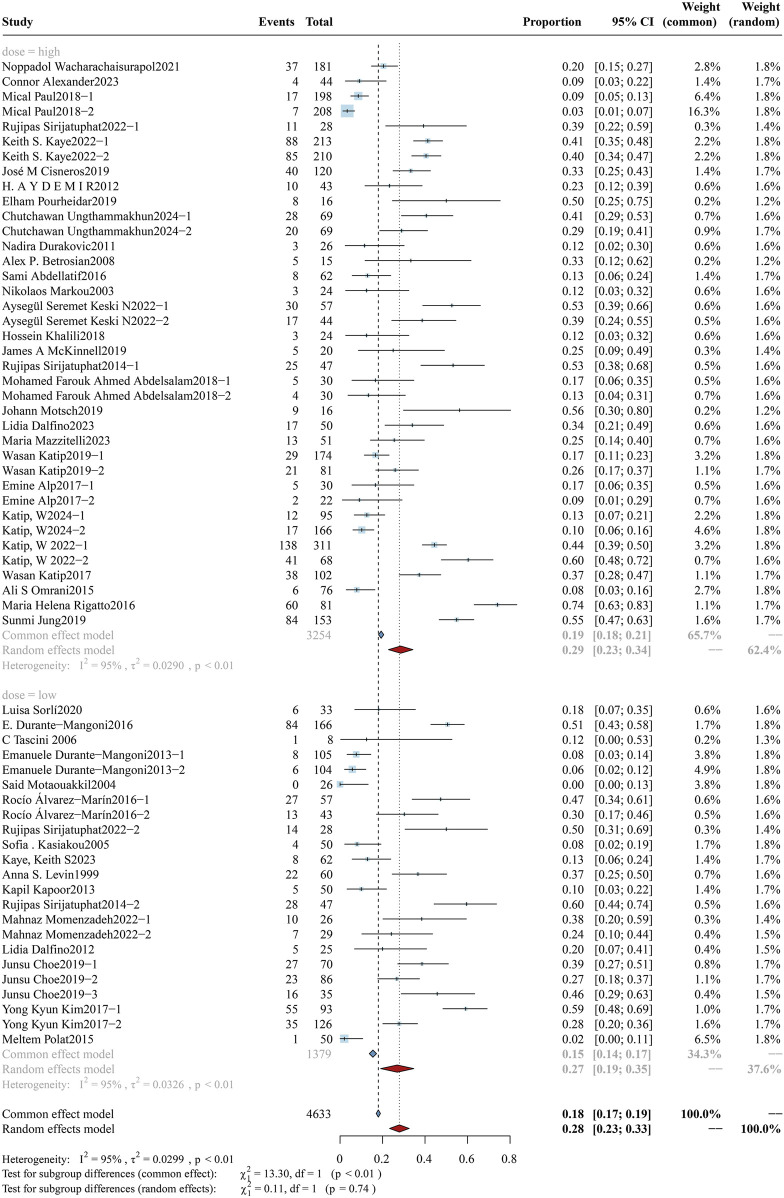
Forest plot of the effect of colistin daily dose (high vs. low) on AKI Incidence The figure presents a subgroup analysis comparing the incidence of colistin-associated acute kidney injury (AKI) between high-dose (≥4.2 mg/kg/day) and low-dose (<4.2 mg/kg/day) colistin regimens.

Different microorganisms exhibit varying susceptibilities to colistin. In this study, we focused on examining the susceptibility of *P. aeruginosa*, drug-resistant Enterobacterales, *K. pneumoniae*, and *A. baumannii* to colistin. Accordingly, the cohort was stratified into five subgroups based on these microbial species. The analysis of mortality rates across subgroups is shown in Figure 6, and the clinical response rates and bacterial eradication rates for each subgroup are presented in Supplementary Material 3 (Figures 7, 8). The study cohort comprised a total of 8,038 patients, categorized as follows: CRAB group (n = 4,686), CRPA group (n = 282), CRPK group (n = 244), CRE group (n = 408), and mixed bacterial infections group (n = 2,418). The mortality rates were as follows: 43.75% (95%CI: 37.67%–49.83%) in the CRAB subgroup, 24.40% (95%CI: 16.58%–32.22%) in the CRPA subgroup, 33.18% (95%CI: 26.37%–39.98%) in the CRKP subgroup, 28.69% (95%CI: 21.11%–36.27%) in the CRE subgroup, and 31.29% (95%CI: 23.70%–38.88%) in the mixed bacterial infections subgroup. A significant difference was found in mortality (p = 0.05), with CRAB-associated infections exhibiting the highest mortality rate, while Patients with CRPA and CRKP infections showed significantly superior clinical response rates relative to other subgroups (p = 0.05), and CRE demonstrated the highest bacterial eradication rate (p < 0.01).

#### 3.3.5 Administration methods: aerosolized plus intravenous colistin vs. intravenous colistin alone

**FIGURE 9:**
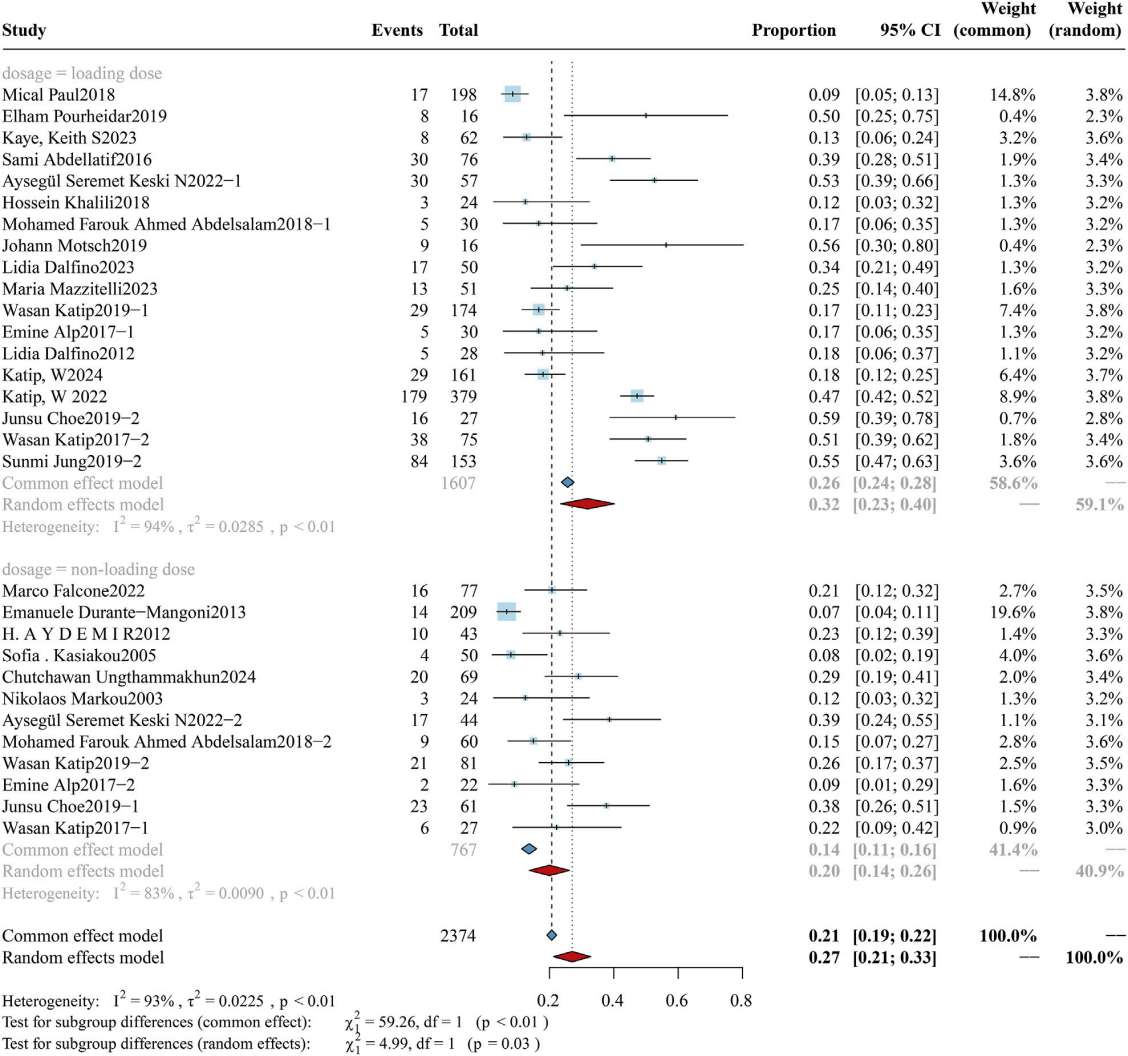
Forest plot of the effect of loading dose on AKI Incidence. The figure shows a subgroup analysis comparing the incidence of colistin-associated acute kidney injury (AKI) between patients who received a loading dose and those who did not.

**FIGURE 10:**
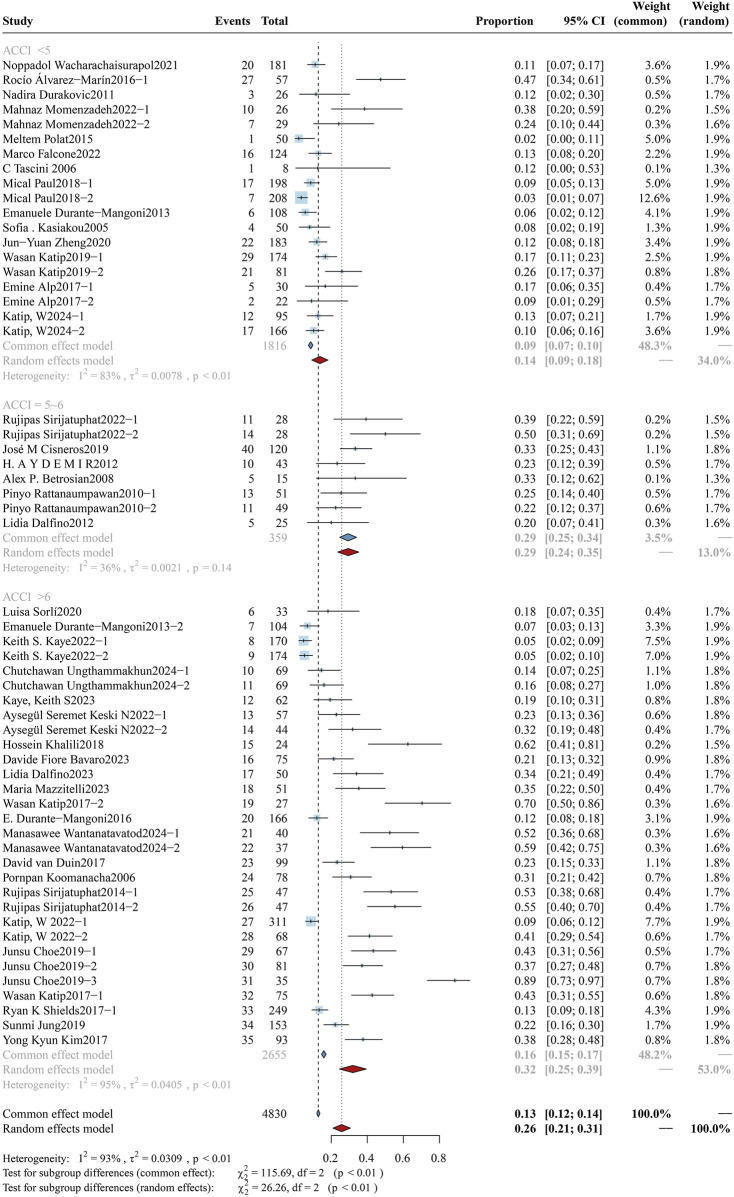
Forest plot of the effect of ACCI on AKI Incidence The figure presents a subgroup analysis evaluating the impact of age-adjusted Charlson Comorbidity Index (ACCI) on the incidence of colistin-associated acute kidney injury (AKI). Studies are stratified into three subgroups based on ACCI scores: ACCI <5, ACCI = 5–6, and ACCI >6.

The study cohort exclusively enrolled patients diagnosed with hospital-acquired or ventilator-associated pneumonia (HAP/VAP), who were categorized into three treatment subgroups: intravenous alone, aerosolized plus intravenous, and aerosolized alone. The cohort comprised a total of 2,887 patients, distributed as follows: 1,841 patients in the intravenous monotherapy group, 713 patients in the combined intravenous-nebulized therapy group, and 333 patients in the nebulized monotherapy group. Analyses of mortality rates among subgroups are illustrated in Figure 7, with corresponding evaluations of clinical efficacy and bacterial eradication rates documented in Supplementary Material 3 (Figures 9, 10). In the subgroup analysis of intravenous alone *versus* aerosolized plus intravenous therapy, the overall mortality rates were: 40.81% (95%CI: 34.56%–47.07%) vs. 32.84% (95%CI: 24.19%–41.48%), clinical response rates were 57.50% (95%CI: 54.70%–60.29%) vs. 64.66% (95%CI: 61.42%–67.90%), and microbiological eradication rates were 54.30% (95%CI: 46.96%–61.65%) vs. 59.56% (95%CI: 49.58%–69.54%). Although clinical differences were observed between the two groups, no statistically significant difference was found (p = 0.09).

### 3.4 Safety outcome

#### 3.4.1 Colistin daily dose

As colistin is typically employed as salvage therapy for CRO infections, it is imperative that colistin be administered early at adequate doses to maximize therapeutic efficacy while simultaneously mitigating the emergence of resistance and minimizing the risk of nephrotoxicity. The study cohort was further stratified into two subgroups: high-dose (n = 3,254) and low-dose (n = 1,379), comprising a total of 4,633 participants. A comparative analysis of AKI incidence between the subgroups is presented in Figure 8. The AKI incidence was 28.55% (95% CI: 22.80%–34.30%) in the high-dose group and 26.92% (95% CI: 19.13%–34.71%) in the low-dose group, with a pooled overall incidence of 27.93% (95% CI: 23.33%–32.53%). No significant difference was found between the subgroups (p = 0.74). In addition, the analysis of the loading dose cohort (Figure 9) revealed that the incidence of colistin-associated AKI was significantly higher in the loading dose subgroup compared with the maintenance dose subgroup [31.83% (95% CI: 23.48%–40.18%) vs. 19.97% (95% CI: 13.76%–26.18%), p = 0.03]. It was concluded that colistin-induced AKI correlates specifically with the administration of loading doses, with no significant association observed with daily dosing regimens.

#### 3.4.2 ACCI

According to the ACCI score, the patients were divided into three subgroups: low-ACCI, moderate-ACCI, and high-ACCI. The analysis results are shown in Figure 10. A total of 4,830 patients were included in this study. The incidence of AKI in the three subgroups was 13.71% (95% CI: 9.23%–18.18%) for the low-ACCI group, 29.43% (95% CI: 23.59%–35.27%) for the moderate-ACCI group, and 31.98% (95% CI: 24.51%–39.46%) for the high-ACCI group respectively. Random effects model analysis showed that ACCI was related to colistin-induced AKI. (p < 0.01).

#### 3.4.3 Co-therapy

**FIGURE 11:**
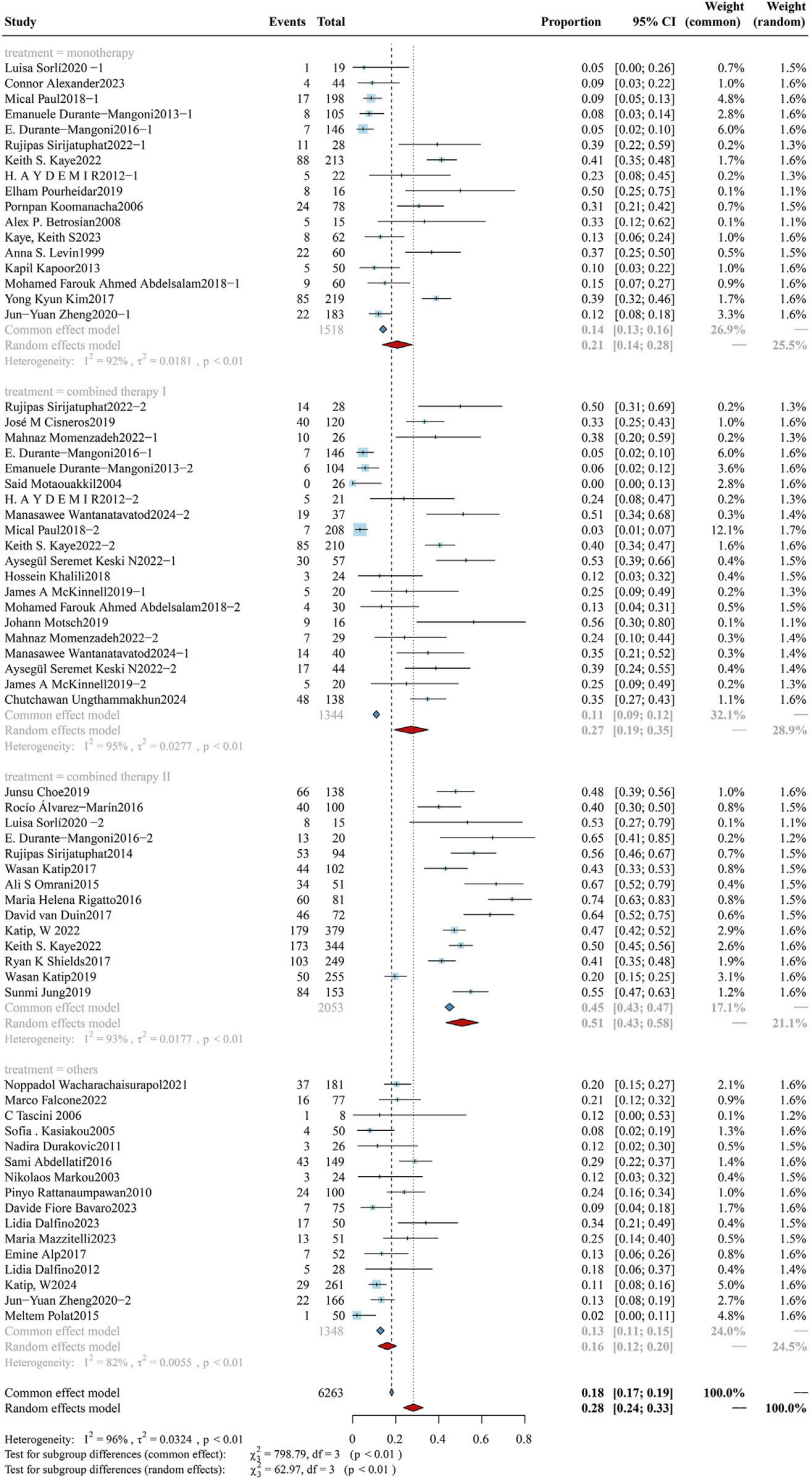
Forest plot of the effect of co-therapy on AKI Incidence. The figure presents a subgroup meta-analysis evaluating the impact of different co-therapy regimens on the incidence of colistin-associated acute kidney injury (AKI). Studies are stratified into four subgroups based on concomitant medications: (1) monotherapy (colistin alone); (2) Combination Therapy Group I—agents with relatively lower nephrotoxicity risk (including carbapenems, tigecycline, β-lactams, and quinolones); (3) Combination Therapy Group II—agents with higher nephrotoxicity risk (including glycopeptides, loop diuretics, and aminoglycosides); and (4) other combination regimens.

In this study cohort, we focused on the impact of concomitant use of colistin with nephrotoxic agents—including vancomycin, aminoglycosides, loop diuretics, and other relevant medications—on the incidence of AKI. A total of 6,263 patients were grouped as follows: the colistin monotherapy group (n = 1,518), Combination Therapy Group I (carbapenems/tigecycline/β-lactams/quinolones) (n = 1,344), Combination Therapy Group II (glycopeptides/loop diuretics/aminoglycosides) (n = 2,053), and others group. The forest plot analysis of the random-effects model is shown in Figure 11. The incidence of AKI was 20.75% (95% CI: 13.80%–27.69%) in the monotherapy group, 27.25% (95% CI: 19.36%–35.14%) in combination group I, and 50.88% (95% CI: 43.33%–58.43%) in combination group II. The results indicate that the concomitant use of colistin with nephrotoxic drugs significantly increases the incidence of AKI (p < 0.01).

#### 3.4.4 Microbial species

**FIGURE 12:**
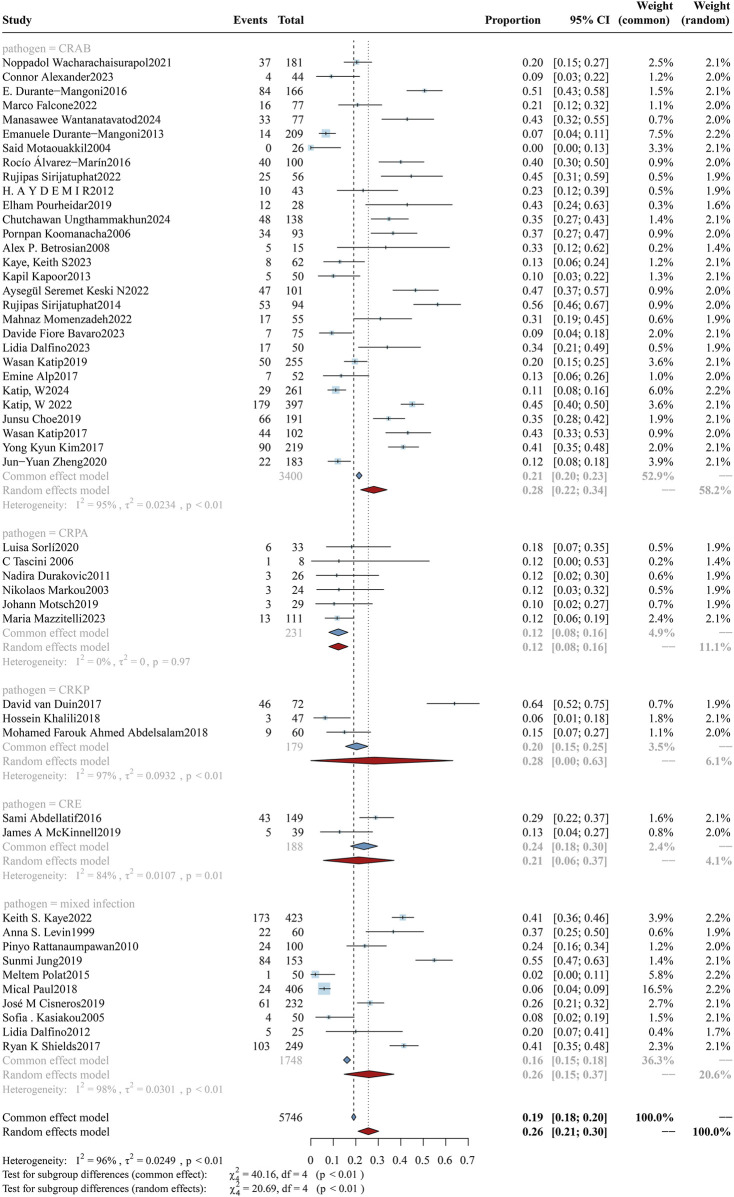
Forest plot of the effect of microbial species on AKI Incidence. The forest plot presents a subgroup analysis of colistin-associated acute kidney injury (AKI) incidence stratified by the causative Gram-negative pathogens. Subgroups include infections caused by carbapenem-resistant *A. baumannii* (CRAB), carbapenem-resistant Enterobacterales (CRE), carbapenem-resistant *K. pneumoniae* (CRKP), carbapenem-resistant *Pseudomonas aeruginosa* (CRPA), and mixed infections involving multiple resistant Gram-negative bacterial species.

The incidence rates of AKI associated with colistin therapy across different microbial infections are comprehensively presented in Figure 12. The cohort comprised 5,746 patients, including 231 cases of CRPA, 3,400 cases of CRAB, 179 cases of CRKP, 188 cases of CRE, and 1,748 cases of mixed bacterial infections. The AKI incidence rates in the five subgroups were 12.27% (95% CI: 8.04%–16.49%), 28.02% (95% CI: 22.17%–33.86%), 28.22% (95% CI: 8.04%–63.17%), 21.30% (95% CI: 5.61%–36.99%), and 25.89% (95% CI: 14.84%–36.94%), respectively. Despite notable heterogeneity, the random-effects model analysis indicated that when colistin was employed for treating *P. aeruginosa* infections, the incidence of colistin-associated AKI was significantly lower compared to infections caused by other microorganisms (p < 0.01).

#### 3.4.5 Administration methods: aerosolized plus intravenous colistin vs. intravenous colistin alone

**FIGURE 13:**
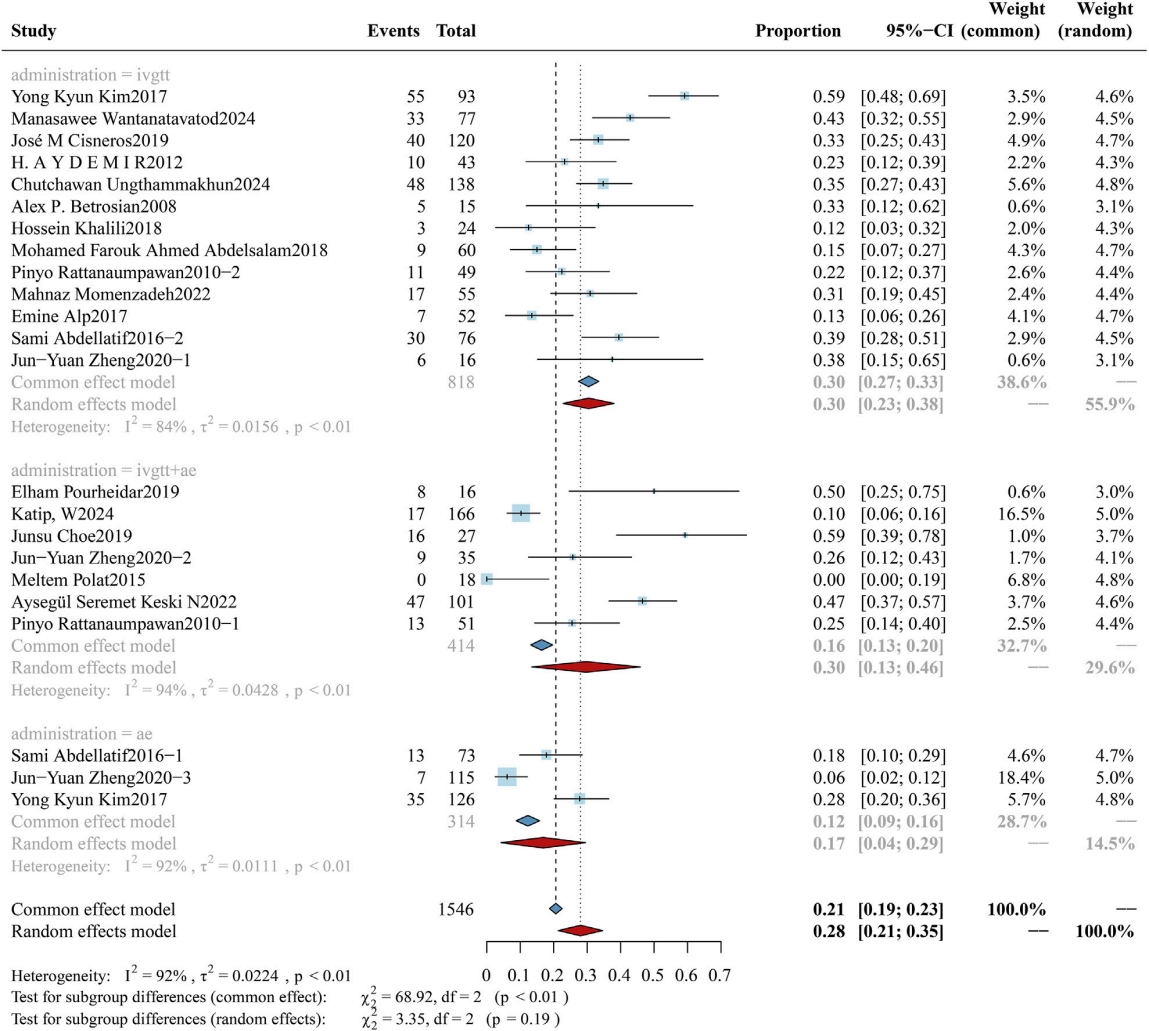
Forest plot of the effect of administration methods on AKI Incidence. The figure presents a subgroup meta-analysis comparing the incidence of colistin-associated AKI across three administration methods: intravenous monotherapy (ivgtt), intravenous-aerosolized therapy (ivgtt + ae), and aerosolized monotherapy (ae).

For critically ill patients with HAP/VAP, colistin combination therapy (aerosolized plus intravenous colistin) demonstrates clinical efficacy against carbapenem-resistant Gram-negative pathogens. The cohort included a total of 1,546 patients with CRO-induced pulmonary infections, comprising 818 cases in the intravenous group, 414 cases in the aerosolized plus intravenous group, and 314 cases in the aerosolized group. The incidence rates of AKI across subgroups were 30.43% (95% CI: 22.80%–38.07%), 29.67% (95% CI: 13.47%–45.87%), and 16.88% (95% CI: 4.25%–29.50%) respectively. Figure 13 demonstrates visually that no statistically significant difference was observed in AKI incidence between the subgroups (p = 0.19).

### 3.5 Bias, sensitivity, and heterogeneity evaluation

The risk of bias in the included observational studies and RCTs was assessed using the Newcastle-Ottawa Scale (NOS) and the Cochrane Risk of Bias Tool, respectively, with the detailed evaluation results provided in Supplementary Material 4. Publication bias was assessed using Egger’s test (see Supplementary Material 5), which indicated substantial heterogeneity across studies. Additionally, to address the significant heterogeneity (I^2^> 75%) observed. Subsequent subgroup analyses and sensitivity analyses (excluding studies one by one; see Supplementary Material 6) were performed to explore sources of heterogeneity and assess the robustness of findings. The multivariate meta-regression analysis (Supplementary Material 7) identified patient age and study type as significant predictors of effect size. Patient age was positively associated with effect size (β = 0.0217, p = 0.0088, 95% CI: 0.0055–0.0379), indicating that effect size increased with advancing age. Moreover, compared with non-randomized studies, RCTs were associated with a significantly smaller effect size (β = −0.7680, p = 0.0005, 95% CI: −1.2029 to −0.3331). In contrast, treatment regimen and continent were not statistically significant predictors in the model. Despite the inclusion of these covariates, residual heterogeneity persisted, suggesting that additional, unmeasured factors may have contributed to the variation in study results.

## 4 Discussion

This meta-analysis systematically evaluated the factors influencing the efficacy and safety of colistin in treating drug-resistant Gram-negative bacterial infections from three dimensions: drugs, host-related factors, and microbial characteristics.

Our findings revealed that the daily dose of colistin showed no statistically significant differences in efficacy outcomes including mortality rates and clinical response rates, which aligns with previous studies (Katip et al., 2017; Alp et al., 2017). On the one hand, pharmacokinetic studies have demonstrated that the 24-h area under the free drug plasma concentration-time curve to minimum inhibitory concentration ratio (fAUC/MIC) serves as the best correlate of antibacterial activity for colistin following intravenous administration of CMS (Couet et al., 2012). However, routine monitoring of fAUC remains clinically impractical due to technical limitations. Notably, Plachouras et al., 2009 found that when maintaining a constant daily CMS dose of 9 MIU, there was no significant difference in peak CMS concentrations between the three-dose regimen (3 MIU every 8 h) and the twice-daily regimen (4.5 MIU every 12 h). In contrast, a single daily administration of the equivalent total dose resulted in a statistically significant elevation of peak plasma concentrations compared to divided-dose regimens. Additionally, the study revealed that colistin exhibited a flatter plasma concentration-time profile compared to CMS, which was consistent with its longer elimination half-life (Plachouras et al., 2009). These findings suggest that, unlike polymyxin B sulfate, the daily dose of CMS shows no statistically significant correlation with clinical outcomes.

Conversely, a loading dose of colistin enables rapid achievement of steady-state concentrations (Css), which may exert a more substantial influence on therapeutic efficacy. Our results, in line with multiple prior studies (Li et al., 2004; Landersdorfer and Nation, 2015; Nazer and Anabtawi, 2017), support the use of an intravenous loading dose in critically ill patients with CRO infections. The study by Martinez et al. (2012) demonstrated that adequate drug exposure at the initiation of therapy is essential to optimizing infection-related clinical outcomes (Martinez et al., 2012). While Kumar et al. (2006) demonstrated in animal models that delayed antimicrobial administration significantly increases mortality (Kumar et al., 2006). It is worth noting that colistin is administered as the prodrug CMS, which predominantly requires renal metabolic activation before being reabsorbed into the systemic circulation to exert its bactericidal effects. This pharmacokinetic pathway suggests that patients’ baseline glomerular filtration rate (GFR) may substantially influence optimal colistin dosing regimens (Gautam et al., 2018). This may explain the absence of a statistically significant difference in therapeutic efficacy between high- and low-dose subgroups observed in our meta-analysis. Other prior studies have demonstrated that colistin exhibits significantly enhanced therapeutic efficacy when administered at elevated doses. Compared with our study, these studies have predominantly enrolled homogeneous patient cohorts restricted to specific infection sites such as burn injuries and HAP/VAP, consequently limiting the generalizability of their findings to broader patient populations (Falagas et al., 2010). In clinical practice, colistin is typically administered using fixed or weight-based dosing regimens without a defined target mean Css. As a result, interpatient variability in Css is substantial (Nation, Velkov, and Li, 2014), and the fractional conversion of CMS to colistin adds another layer of unpredictability (Grégoire et al., 2014).

Similar to findings on efficacy outcome, existing research remains inconclusive regarding the association between daily colistin dosing and AKI incidence. Our analysis demonstrated no statistically significant correlation between colistin daily dosing and AKI occurrence (p = 0.74), which is consistent with previous reports (Dalfino et al., 2012; Kwon et al., 2015; Wilkinson et al., 2017). However, conflicting evidence exists as several studies suggested a dose-dependent increase in AKI risk with high-dose colistin regimens (Vicari et al., 2013; Benattar et al., 2016; Omrani et al., 2015). These discrepancies likely stem from heterogeneity in patient populations, inconsistencies in AKI diagnostic criteria (KDIGO vs. RIFLE), and variation in controlling for confounders such as concurrent nephrotoxic medications. Furthermore, a significant increase in the incidence of AKI is observed only when the average Css of colistin exceeds 2 mg/L. However, achieving colistin Css levels above this threshold proves clinically challenging in most patients, as such concentrations are rarely attained without administering an initial loading dose (Couet et al., 2012). The heterogeneity in patients’ baseline renal function partially contributes to the occurrence of colistin-associated AKI. The current international consensus recommends maintenance doses based on individuals with normal renal function; therefore, our subgroup definitions for high- and low-dose were grounded on this baseline. In patients with impaired renal function, unchanged daily dosing may inadvertently result in plasma concentrations exceeding 2 mg/L, which might enhance bactericidal activity but also increase nephrotoxicity. Therefore, in our low-dose subgroup, the actual Css in renally impaired patients may have been higher than theoretical predictions, confounding the observed associations. Therapeutic drug monitoring (TDM) may help optimize exposure and minimize toxicity, but more research is needed to determine the most appropriate patient populations and implementation strategies for TDM in colistin therapy. Furthermore, concomitant medication regimens also partially contribute to the occurrence of colistin-associated AKI. In conclusion, the loading dose of colistin is associated with both therapeutic efficacy and the risk of colistin-associated AKI, highlighting the delicate balance clinicians must navigate between maximizing antibacterial activity and minimizing nephrotoxicity.

The influence of patient’s comorbidities and physiological status on the therapeutic efficacy of colistin underscores the importance of individualized treatment strategies in clinical practice. Multiple studies have demonstrated that advanced age and obesity are independent risk factors influencing both the efficacy and safety of colistin therapy, although the specific thresholds for age and body weight associated with heightened risk vary across studies (Gauthier et al., 2012; Tuon et al., 2014). Nevertheless, the high proportion of missing values in recorded body weights among the analyzed cohort precluded further investigation into potential weight-associated effects. Renal impairment may significantly hinder colistin metabolism and clearance, increasing the risk of adverse effects, particularly nephrotoxicity (Bk et al., 2021). Additionally, immunocompromised conditions resulting from diabetes mellitus and hematologic malignancies may impair host immune responses and consequently reduce antimicrobial efficacy (Gunay et al., 2020). Based on prior studies, we employed the ACCI to comprehensively evaluate both chronological age and comorbidity burden of patient baseline status. Our findings revealed statistically significant differences in overall mortality and clinical efficacy rates between the low-ACCI group (score <5), moderate-ACCI group (score from 5 to 6), and high-ACCI group (score >6) (p < 0.01), supporting ACCI as a risk factor affecting the efficacy and safety of colistin.

Previous studies have demonstrated that the prevalence of heteroresistance to polymyxins exceeds 14% (Hawley et al., 2007), and combination therapy may mitigate bacterial heteroresistance through synergistic mechanisms. Results of our study demonstrated that the combination of colistin with carbapenems and quinolones significantly reduced mortality rates, improving clinical response and bacterial eradication rates. For infections caused by CRAB, including HAP/VAP and bloodstream infections, combination regimens of colistin with rifampicin (Durante-Mangoni et al., 2013), fosfomycin (Sirijatuphat and Thamlikitkul, 2014), or sulbactam (Ungthammakhun et al., 2024) have shown favorable therapeutic efficacy. These combination therapies exhibited significant advantages over colistin monotherapy, particularly in terms of bacterial eradication rates. In the context of intra-abdominal infections, the combination therapy of colistin and fosfomycin demonstrates comparable therapeutic advantages. Similarly, in the context of UTI and skin infections, colistin combined with fosfomycin has demonstrated comparable therapeutic advantages, which aligns consistently with the findings of our study (Durante-Mangoni et al., 2013). In our study, the colistin-fosfomycin subgroup demonstrated a notably elevated bacterial eradication rate (98.17%, 95%CI: 96.07%–100.00%), which potentially attributed to the inclusion of urinary tract infection patients where both colistin and fosfomycin achieve high urinary concentrations (Parker et al., 2015). Whereas, the results of the largest RCT to date (the AIDA study) reported no statistically significant differences between the colistin monotherapy group and the colistin-meropenem combination therapy group in clinical failure rates (79% vs. 73%; p = 0.17), 28-day mortality (43% vs. 45%; p = 0.78), or bacterial failure rates (31% vs. 35%; p = 0.49; Paul et al., 2018). Despite such discrepancies, current international guidelines recommend the use of colistin in combination with other antimicrobials for the treatment of multidrug-resistant Gram-negative infections (Tsuji et al., 2019).

On the other hand, our findings demonstrate that colistin administration in combination with nephrotoxic agents, such as glycopeptides, aminoglycoside antibiotics, and loop diuretics, significantly increases the incidence of AKI. These concomitant medications were identified as independent risk factors for colistin-associated AKI (Petrosillo et al., 2014; Temocin et al., 2015). Although most studies in this subgroup were observational and methodologically limited, the growing body of consistent evidence has strengthened the overall quality of this association. This conclusion is further supported by a comprehensive meta-analysis of 237 studies involving more than 35,000 patients (Wagenlehner et al., 2021). While prior studies primarily focused on the safety profile of colistin-based combination regimens, further research is warranted to assess the impact of specific combinations on the development of colistin resistance. Furthermore, future investigations should emphasize exploring how different anatomical sites of infection influence therapeutic outcomes in combined antimicrobial interventions.

The variation in treatment outcomes across microbial species could potentially stem from differential intrinsic resistance profiles to colistin among microorganisms. The results of our study demonstrated a statistically significant difference in overall mortality rates across various bacterial infections (p < 0.01). Specifically, CRAB infections were associated with the highest case fatality rate (43.75%, 95%CI: 37.67%–49.83%), significantly higher than the morality (28.69%, 95%CI: 21.11%–36.27%) observed in CRE infections. Furthermore, the bacterial clearance rate was significantly higher in the CRE subgroup compared to other subgroups, which may be attributed to their lower resistance to colistin. However, the limited number of studies in this subgroup, all observational studies focusing on BSI and UTI, has resulted in a downgrading of the evidence quality. Results from a large-scale international RCT demonstrated that infections caused by CRAB were associated with high mortality and clinical failure rates (Kaye et al., 2022). In a large systematic review on urinary tract infections, the resistance rate of *P. aeruginosa* to colistin (3.8%) was lower than that of *A*. *baumannii* (4.6%), and in this study, the resistance rate of *P. aeruginosa* to colistin (7.2%) was also lower than that of *A*. *baumannii* (10.0%) in a cross-sectional study of local district hospitals (Requena-Cabello et al., 2024). These studies might explain the lower mortality rate of *P. aeruginosa* than that of *A*. *baumannii* in our study. Compared with *P. aeruginosa*, *A. baumannii* tends to exhibit higher antimicrobial resistance, which may be related to its outer membrane protein expression, biofilm formation ability, and the presence of plasmid-mediated multidrug resistance genes. The primary bactericidal mechanism of colistin involves the interaction of its cationic nonribosomal lipopeptide with lipid A, destabilizing the outer membrane, increasing permeability, and ultimately leading to cell death. In *A. baumannii*, a distinctive resistance mechanism—uncommon among other Gram-negative bacteria—involves spontaneous mutations in the lipid A biosynthesis genes (*lpxA*, *lpxC*, or *lpxD*), which abolish lipopolysaccharide (LPS) or lipid A production and thereby remove the drug’s target. Reduced availability of essential cofactors for LPS synthesis, such as biotin, can further diminish susceptibility to polymyxins (Kyriakidis et al., 2021). Mutations in the PmrA–PmrB two-component regulatory system also contribute to resistance: Adams et al. (2009) identified alterations in both *pmrA* and *pmrB* in resistant strains (Adams et al., 2009), while Sun et al. (2020) reported two novel mutations, *pmrA* ^I13M^ and *pmrB* ^Q270P^, associated with colistin resistance in *A. baumannii* (Sun et al., 2020).

Furthermore, the site of infection, geographical variations, disease severity, and baseline patient characteristics substantially influence clinical outcomes (Papst et al., 2018; Wang et al., 2023). CRE infections are frequently observed in patients with BSI and complicated UTI, whereas CRAB is more commonly associated with pneumonia. Bacterial clearance rates in invasive BSI and complicated UTI are typically higher than those in pulmonary or intracranial infections. Furthermore, the average Css of colistin is significantly elevated in serum compared to its concentrations in pulmonary and cerebral tissue (Boisson et al., 2014). Additionally, existing studies have largely failed to adequately distinguish between colonizing bacteria and invasive bacteria, which constitutes another critical contributing factor to the observed differences in treatment efficacy among various drug-resistant bacterial strains. Our findings also demonstrated a significantly lower incidence of AKI in the *P. aeruginosa* infection subgroup compared to other subgroups. However, due to substantial heterogeneity among studies within this subgroup, the certainty of evidence remains limited. Although several pathogen-focused RCTs have been conducted in recent years (Dl et al., 2020), these investigations pose considerable challenges, with most studies demonstrating limited patient recruitment and substantial heterogeneity across trial designs.

Due to suboptimal distribution characteristics, colistin concentrations in pulmonary tissue may be inadequate. Aerosolized administration has been shown to enhance drug concentration in the epithelial lining fluid of the lungs (Boisson et al., 2014). However, the current research remains controversial, with some studies demonstrating that intravenous adjunctive aerosolized colistin may provide additional therapeutic benefits for patients with CRO-associated pneumonia (Pérez-Pedrero et al., 2011; Moradi Moghaddam et al., 2019). Other studies have reported lower mortality rates in the intravenous adjunctive aerosolized group, while no statistically significant difference in mortality was observed between the two groups (Almangour et al., 2021; Kofteridis et al., 2010). Several clinical guidelines currently recommend intravenous adjuvant aerosolized for the treatment of CRO-associated pneumonia, although as a weak recommendation supported by low-quality evidence (Rello et al., 2017; Tsuji et al., 2019). Further studies have indicated there is no significant difference in hospital mortality (p = 0.63) or ICU mortality (p = 0.92) between combination intravenous-aerosolized therapy and intravenous infusion alone, based on multivariate regression analysis adjusting for age and comorbidities (Kofteridis et al., 2010; Korbila et al., 2010). Despite discrepancies in mortality outcomes, most clinical studies have demonstrated improved bacterial eradication and clinical response rates with adjunctive aerosolized therapy. In our analysis, bacterial clearance and clinical response rates were higher in the combination therapy group (56.02% vs. 45.56% and 62.71% vs. 48.19%, respectively), although these differences did not reach statistical significance. Most studies incorporated into this subgroup analysis consisted of observational designs with limited sample sizes and significant inter-study variability.

Although aerosolized colistin appears to be a better option for adjunctive therapy, there is a relative lack of high-quality RCTs investigating the different administration routes of colistin, and thus its exact role in treating CRO-induced pulmonary infections remains unclear (Tumbarello et al., 2013). In terms of safety assessment, existing evidence from multiple clinical studies suggests that intravenous adjunctive aerosolized colistin is not associated with an elevated risk of nephrotoxicity (specifically colistin-induced AKI), a conclusion corroborated by our observational data (Choe et al., 2019; Almangour et al., 2021). The relatively low dose of aerosolized colistin administration demonstrated minimal impact on the average Css of colistin (Karnik et al., 2013; Boisson et al., 2014), and consequently, no significant difference in colistin-associated AKI incidence was observed between the two administration methods (Gkoufa et al., 2022). Furthermore, although standalone aerosolized colistin monotherapy may improve local microbiological outcomes, it does not significantly enhance clinical prognosis in patients with HAP/VAP. Conversely, this approach has been associated with a marked increase in the incidence of tracheal spasms. Therefore, colistin is not recommended for standalone aerosolization in clinical practice (Zhang et al., 2023). Future research should prioritize investigations into the pharmacokinetic characteristics of colistin in pulmonary tissues to optimize dosing strategies.

This study has several limitations: (1) Significant heterogeneity exists among the included studies, encompassing both observational studies and RCTs. Variations in sample sizes and experimental designs may compromise the robustness of our conclusions. Subgroup analyses did not always adjust for baseline differences, potentially confounding intergroup comparisons. (2) Incomplete data on certain influencing factors limited to more in-depth analyses. Specifically, missing weight records for some patients constrained our ability to comprehensively assess weight-related impacts on pharmacokinetic parameters. Although we implemented body weight-based dose conversions and minimized weight-related variations in plasma drug concentrations through normalization procedures, residual uncertainties persist regarding the precise characterization of concentration-efficacy and concentration-safety relationships. Contrary, this conversion approach may introduce additional measurement errors during dose equivalency calculations. Furthermore, As the original studies did not systematically report drug-resistance gene data, this meta-analysis could not assess this factor. Future studies should prioritize analyzing resistance mechanisms. (3) The included studies exhibited heterogeneity in the definitions of outcome measures, which may have influenced our analytical outcomes to some extent. For example, diagnostic criteria for AKI varied significantly across studies, and the methodological adjustments required to reconcile these differing definitions could potentially introduce measurement errors. Similarly, inconsistencies were observed in mortality assessments, with studies reporting disparate endpoints such as 14-day, 28-day, or 30-day mortality rates. These variations in temporal parameters for mortality evaluation further complicated comparative analyses.

Despite certain limitations, this meta-analysis highlights the importance of a comprehensive, individualized approach that integrates host factors, microbial characteristics, and pharmacological considerations in colistin therapy. To the best of our knowledge, this study represents the most comprehensive investigation to date into the factors influencing the efficacy and safety of colistin. Compared with previous meta-analyses, our study further enhanced the methodological rigor by excluding case reports during study selection, thereby elevating the quality hierarchy of incorporated evidence. Furthermore, our findings highlight the necessity for personalized therapeutic strategies and rigorous renal function monitoring in clinical practice, providing evidential support for optimizing colistin-based regimens and informing future clinical guidelines.

## 5 Conclusion

Our results suggest that the colistin loading dose, ACCI score, combination therapy, and microbial factors may significantly influence its efficacy in treating drug-resistant Gram-negative infections. Specifically, administration of a colistin loading dose, lower ACCI scores, and combination therapy with carbapenem antibiotics were associated with improved treatment success rates, and infections caused by CRE exhibited higher bacterial clearance rates. Conversely, infections caused by *A. baumannii* might adversely affect therapeutic outcomes. Loading dose, concomitant administration of nephrotoxic medications, ACCI, and microbial factors were identified as risk factors for colistin-associated nephrotoxicity. These findings offer preliminary insights into the contentious factors influencing colistin therapy, warrant cautious interpretation, and underscore the need for future studies to better define patient subgroups, standardize treatment regimens, and evaluate outcomes to clarify these interactions and inform individualized treatment.

## Data Availability

The original contributions presented in the study are included in the article/Supplementary Material, further inquiries can be directed to the corresponding author.
