# Supplementary material for: Factors affecting the effectiveness and safety of colistin in treating drug-resistant gram-negative bacterial infections: a meta-analysis
Source: Front Pharmacol. 2025 Oct 29;16:1625595. doi: 10.3389/fphar.2025.1625595 (PMC12605452; doi:10.3389/fphar.2025.1625595)

Table 1. Results of multivariate meta-regression analysis

| Moderator | Estimate | SE | 95% CI | p-value |
| --- | --- | --- | --- | --- |
| course of treatment | -0.0131 | 0.0141 | [-0.0408, 0.0145] | 0.3526 |
| Continent |  |  |  |  |
| Africa | 0.4471 | 0.7808 | [-1.0833, 1.9775] | 0.5669 |
| Asia | 0.0064 | 0.3758 | [-0.7303, 0.7430] | 0.9864 |
| Europe | -0.2966 | 0.3834 | [-1.0480, 0.4548] | 0.4391 |
| North America (reference) | - | | | |
| Study type |  |  |  |  |
| RCT | -0.768 | 0.2219 | [-1.2029, -0.3331] | **0.0005** |
| Observational research (reference) | - | | | |
| Age | 0.0217 | 0.0083 | [0.0055, 0.0379] | **0.0088** |

Figure 1. Boxplot of study type covariates


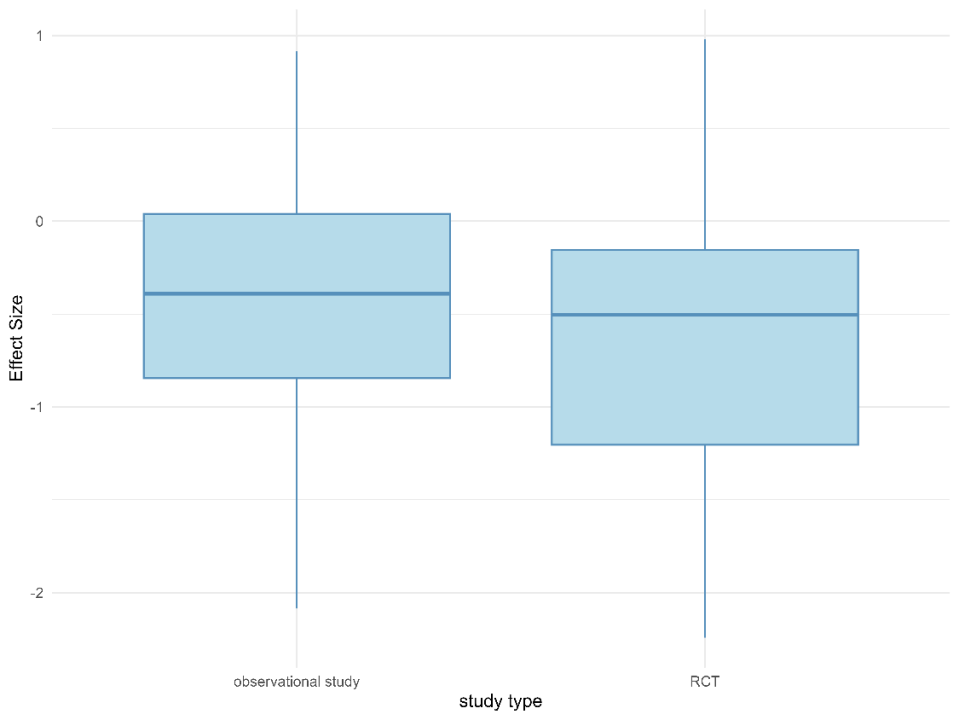


Figure 2. Boxplot of continental covariates


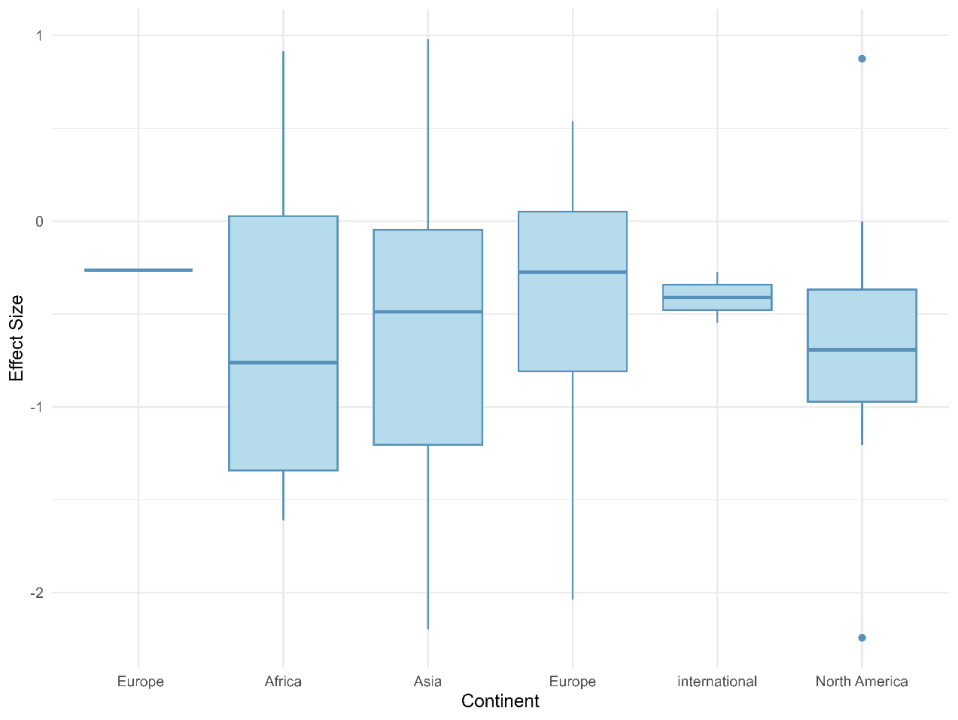


Figure 3. Bubble plot of age covariate


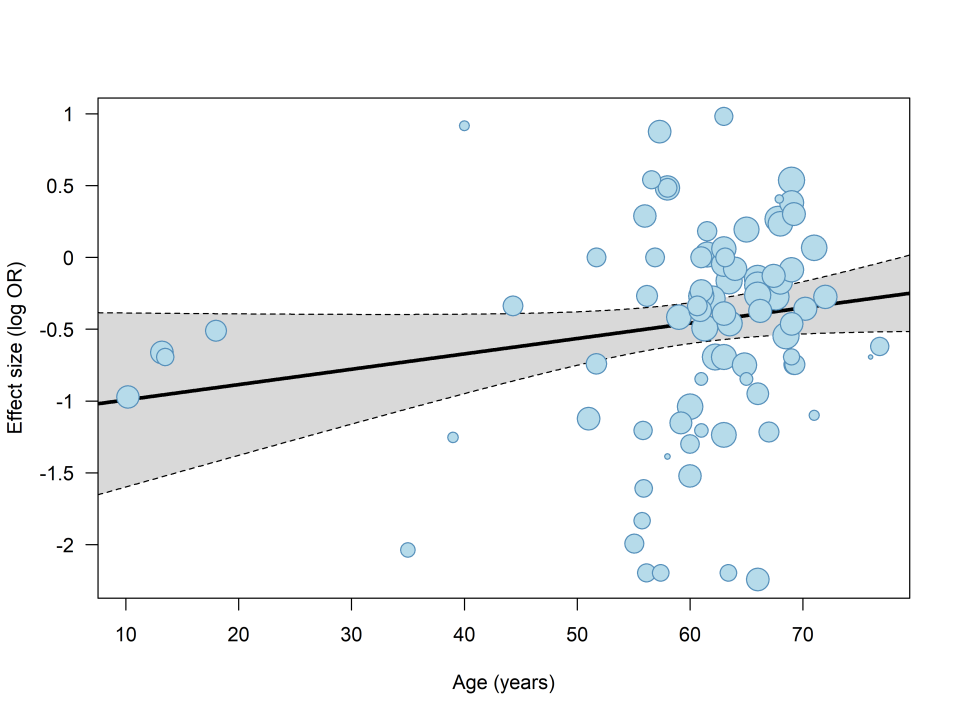


Figure 4. Bubble plot of course of treatment
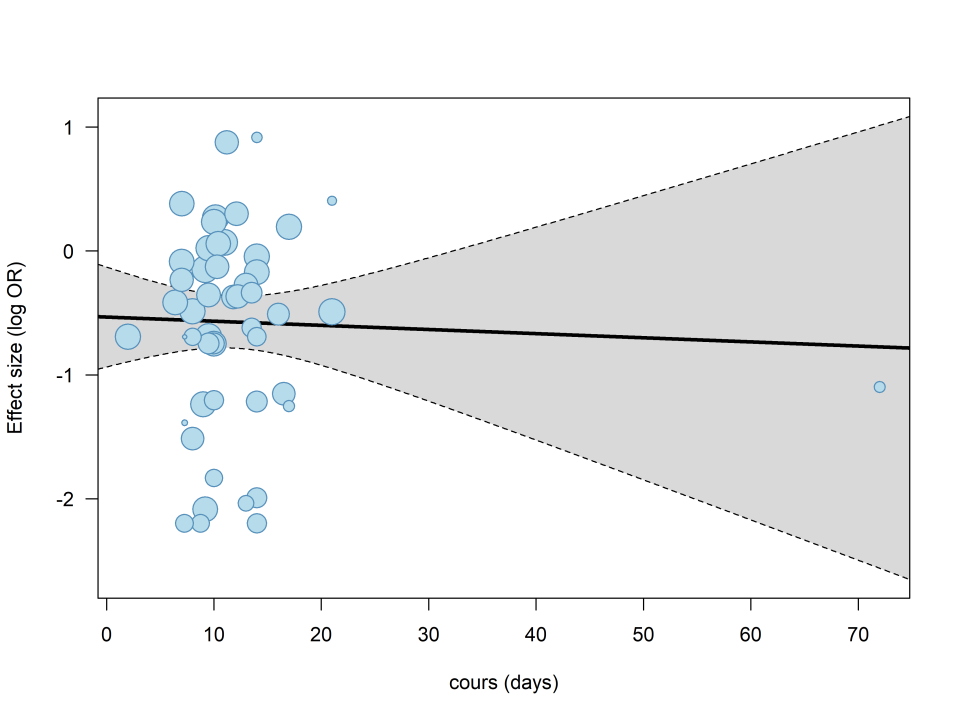

Supplement: Supplementary file 1 [file DataSheet1.zip › Supplementary/Supplementary Material 7. multivariate meta-regression analysis.docx]
