# Supplementary material for: Factors affecting the effectiveness and safety of colistin in treating drug-resistant gram-negative bacterial infections: a meta-analysis
Source: Front Pharmacol. 2025 Oct 29;16:1625595. doi: 10.3389/fphar.2025.1625595 (PMC12605452; doi:10.3389/fphar.2025.1625595)
Supplement: Supplementary file 1 [file DataSheet1.zip › Supplementary/Supplementary Material 3-Forest plot of the clinical response and bacterial eradication rates.docx]

Figure1.Forest plot of the effect of colistin daily dose (high vs low) on clinical response rate

Figure2. Forest plot of the effect of colistin daily dose (high vs low) on bacterial eradication rate


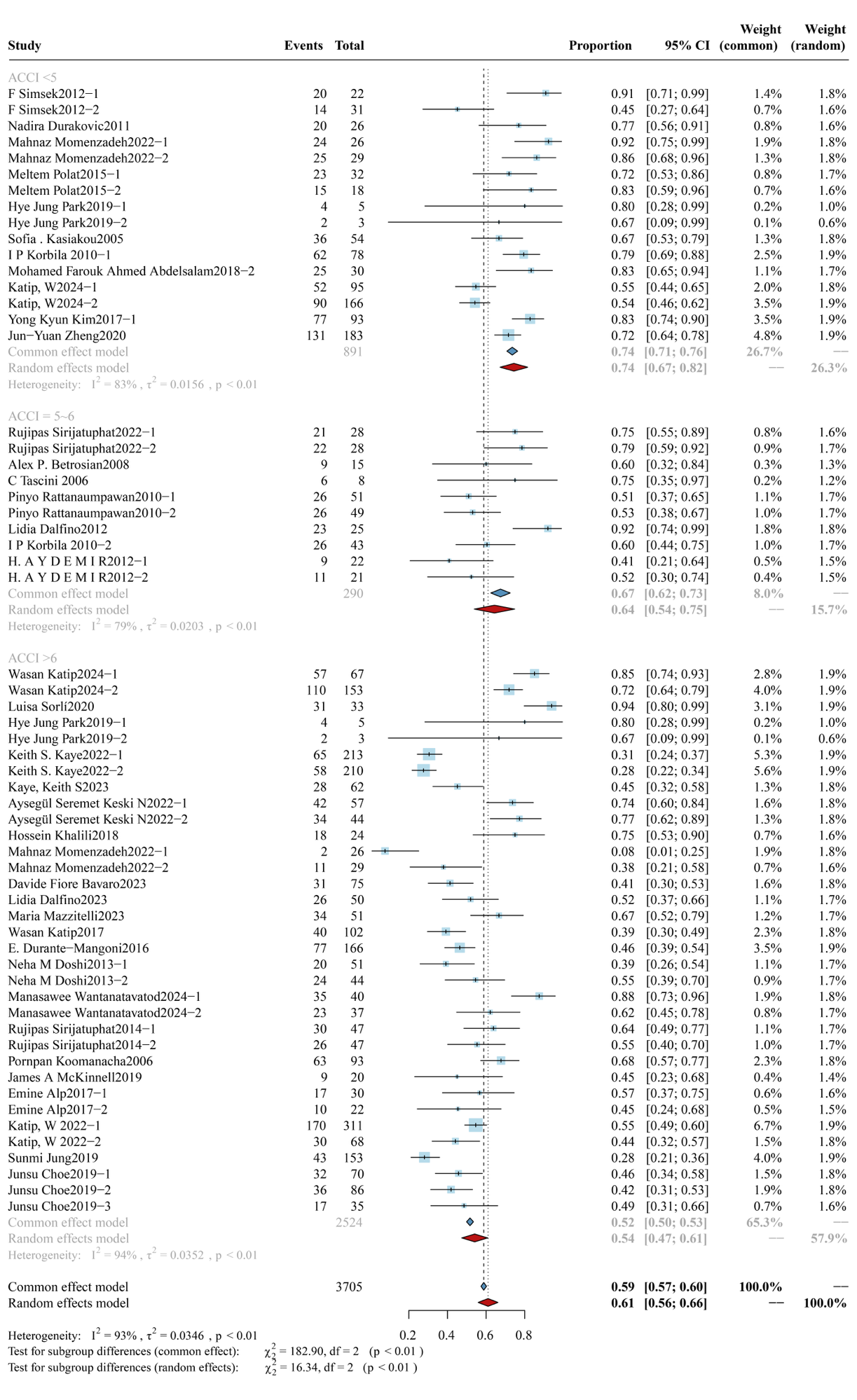


Figure3.Forest plot of the effect of ACCI on clinical response rate
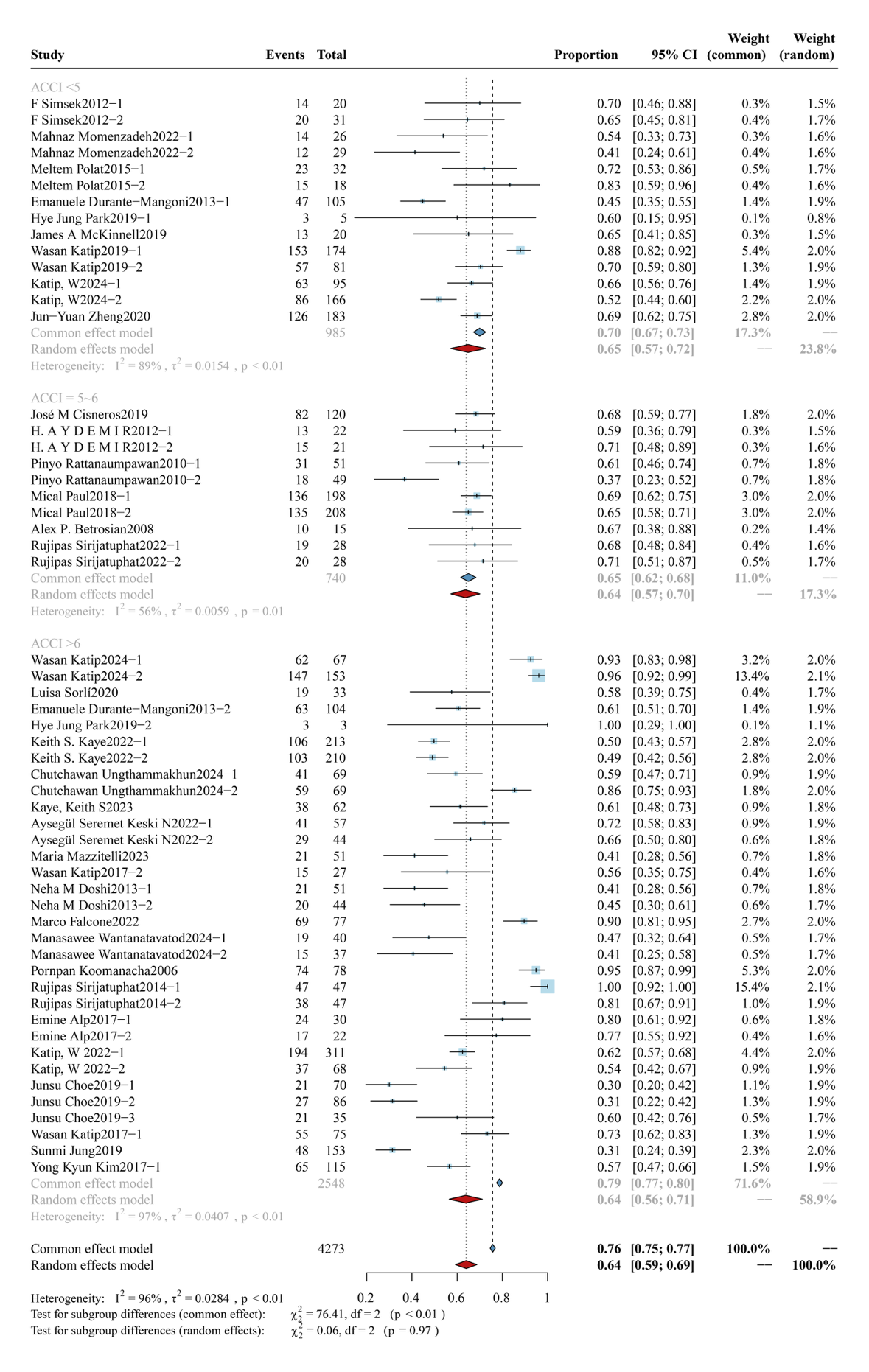


Figure4. Forest plot of the effect of ACCI on bacterial eradication rate

Figure5. Forest plot of the effect of co-therapy on clinical response rate

Figure6. Forest plot of the effect of co-therapy on bacterial eradication rate

Figure7. Forest plot of the effect of microbial species on clinical response rate

Figure8. Forest plot of the effect of microbial species on bacterial eradication rate

Figure9. Forest plot of the effect of administration methods on clinical response rate

Figure10. Forest plot of the effect of administration methods on bacterial eradication rate
