# Supplementary material for: Factors affecting the effectiveness and safety of colistin in treating drug-resistant gram-negative bacterial infections: a meta-analysis
Source: Front Pharmacol. 2025 Oct 29;16:1625595. doi: 10.3389/fphar.2025.1625595 (PMC12605452; doi:10.3389/fphar.2025.1625595)
Supplement: Supplementary file 1 [file DataSheet1.zip › Supplementary/Supplementary Material 2- Summary of included studies.docx]

**Supplementary File 2 Summary of included studies**

| ID | Study | Region | Study type | Patients  number | Dose (CBA)mg/kg/d | aCCl | Results | | | |
| --- | --- | --- | --- | --- | --- | --- | --- | --- | --- | --- |
|  |  |  |  |  |  |  | mortality | Clinic ally effective | Bacterial clearance | AKI |
| 1 | Noppadol Wacharachaisurapol2021 | Thailand | retrospective study | 181 | 5.1 | 1.38 | 1 | 0 | 0 | 1 |
| 2 | Wasan Katip2024 | Thailand | retrospective study | 220 | 4.2 4.2 | arm1:7.28 arm2:7.51 | 1 | 1 | 1 | 0 |
| 3 | Luisa Sorlí2020 | Spain | Prospective cohort study | 33 | 2.31 | 7.9 | 0 | 1 | 1 | 1 |
| 4 | Connor Alexander2023 | USA | Retrospective case-control | 55 | 4.52 | NA | 0 | 1 | 0 | 1 |
| 5 | E. Durante-Mangoni2016 | Italy | Prospective cohort study | 166 | 2.78 | 4.93 | 1 | 1 | 0 | 1 |
| 6 | Neha M Doshi2013 | USA | retrospective study | 95 | 3.7 4 | arm1:3.37 arm2:5.1 | 1 | 1 | 1 | 0 |
| 7 | Marco Falcone2022 | Italy | retrospective study | 124 | NA | 6 | 1 | 0 | 1 | 1 |
| 8 | C Tascini 2006 | Italy | retrospective study | 8 | 0.92 | 6 | 1 | 1 | 0 | 1 |
| 9 | Manasawee Wantanatavatod2024 | Thailand | RCT | 77 | NA | arm1:4.59 arm2:5.17 | 1 | 1 | 1 | 1 |
| 10 | Sarah M McLeod2024 | Multicentric | RCT | 125 | NA | NA | 1 | 1 | 1 | 0 |
| 11 | F Simsek2012 | India | Retrospective case-control | 51 | 2.5-5 2.5-5 | arm1:2.6 arm2:2.6 | 1 | 1 | 1 | 0 |
| 12 | Mical Paul2018 | Israel、Greece and Italy | RCT | 406 | 4.2 4.2 | arm1:5 arm2:5 | 1 | 1 | 1 | 1 |
| 13 | Emanuele Durante-Mangoni2013 | Italy | RCT | 209 | 2.8 2.8 | arm1:5.59 arm2:7.54 | 1 | 0 | 1 | 1 |
| 14 | Said Motaouakkil2004 | Morocco | retrospective study | 26 | 2 | NA | 0 | 1 | 0 | 1 |
| 15 | Rocío Álvarez-Marín2016 | Spain | Prospective cohort study | 100 | 2.8 2.8 | arm1:2 arm2:4 | 1 | 0 | 0 | 1 |
| 16 | Rujipas Sirijatuphat2022 | Thailand | RCT | 56 | 4.3 ± 2.1 4.0 ± 1.7 | arm1:5.57 arm2:5.32 | 1 | 1 | 1 | 1 |
| 17 | Hye Jung Park2019 | Korea | RCT | 8 | 1.7 | arm1:6 arm2:7.5 | 1 | 1 | 1 | 0 |
| 18 | Keith S. Kaye2022 | Multicentric | RCT | 423 | 5 5 | arm1:8 arm2:8 | 1 | 1 | 1 | 1 |
| 19 | David van Duin2017 | USA | Prospective cohort study | 137 | NA | 6 | 1 | 0 | 0 | 1 |
| 20 | Demosthenes Makris2018 | Greece | RCT | 39 | 4.2 4.2 | NA | 1 | 1 | 1 | 0 |
| 21 | José M Cisneros2019 | Multicentric | RCT | 232 | 4.2 | 3.6 | 1 | 0 | 1 | 1 |
| 22 | H. A Y D E M I R2012 | Turkey | RCT | 43 | 4.2 4.2 | arm1:4.68 arm2:3.19 | 1 | 1 | 1 | 1 |
| 23 | Sofia . Kasiakou2005 | Greece | retrospective study | 50 | 1.4 | 4.6 | 1 | 1 | 0 | 1 |
| 24 | Atousa Hakamifard2023 | Iran | RCT | 55 | NA | NA | 1 | 0 | 0 | 0 |
| 25 | Elham Pourheidar2019 | Iran | RCT | 28 | 4.2 | NA | 1 | 1 | 1 | 1 |
| 26 | Keith S. Kaye2020 | USA | RCT | 23 | NA | NA | 1 | 1 | 0 | 0 |
| 27 | Chutchawan Ungthammakhun2024 | Thailand | RCT | 138 | 4.2 4.2 | arm1:7.07 arm2:7.14 | 1 | 0 | 1 | 1 |
| 28 | Pornpan Koomanacha2006 | Thailand | RCT | 93 | NA | 3.73 | 1 | 1 | 1 | 1 |
| 29 | Nadira Durakovic2011 | Croatia | retrospective study | 26 | 4.2 4.2 | arm1:2 arm2:2 | 1 | 1 | 0 | 1 |
| 30 | Alex P. Betrosian2008 | Greece | Prospective cohort study | 15 | 4.2 | 3.7 | 1 | 1 | 1 | 1 |
| 31 | Kaye, Keith S2023 | USA | RCT | 125 | 2.5 | 8 | 1 | 1 | 1 | 1 |
| 32 | Sami Abdellatif2016 | Tunisia | RCT | 149 | 4.2 | NA | 1 | 1 | 1 | 1 |
| 33 | Alessandro Russo 2020 | Italy | Prospective cohort study | 151 | NA | NA | 1 | 0 | 0 | 0 |
| 34 | Anna S. Levin1999 | Brazil | Retrospective case-control | 60 | 2.5 | NA | 0 | 1 | 0 | 1 |
| 35 | Kapil Kapoor2013 | India | retrospective study | 50 | 1.7-2.5 | NA | 1 | 1 | 0 | 1 |
| 36 | Nikolaos Markou2003 | Greece | retrospective study | 24 | 4.2 | NA | 1 | 1 | 0 | 1 |
| 37 | Ayşegül Seremet Keski N2022 | Turkey | retrospective study | 101 | 4.2 4.2 | arm1:9 arm2:9 | 1 | 1 | 1 | 1 |
| 38 | Hossein Khalili2018 | Iran | RCT | 47 | 4.2 | 7 | 1 | 1 | 0 | 1 |
| 39 | Abdul Ghafur2017 | India | retrospective study | 153 | 4.2 4.2 | NA | 1 | 1 | 1 | 0 |
| 40 | James A McKinnell2019 | USA | RCT | 39 | 5 | 5.6 | 1 | 1 | 1 | 1 |
| 41 | Rujipas Sirijatuphat2014 | Thailand | RCT | 94 | 4.4 ± 1.5 4.0 ± 1.5 | arm1:5.23 arm2:4.94 | 1 | 1 | 1 | 1 |
| 42 | Mohamed Farouk Ahmed Abdelsalam2018 | Egypt | RCT | 60 | 4.2 4.2 | arm1:3.6 arm2:3.2 | 1 | 1 | 0 | 1 |
| 43 | K. S. Bharathi2022 | USA | RCT | 98 | 3 | NA | 1 | 1 | 1 | 0 |
| 44 | Pinyo Rattanaumpawan2010 | Thailand | RCT | 100 | NA | arm1:5.7 arm2:4.65 | 1 | 1 | 1 | 1 |
| 45 | Johann Motsch2019 | Germany | RCT | 31 | 4.2 | NA | 1 | 1 | 0 | 1 |
| 46 | Yael Zak-Doron2018 | Greece | RCT | 209 | NA | 5 | 1 | 0 | 0 | 0 |
| 47 | T. Amat2018 | Spain | retrospective study | 118 | 4.2 1.4 | arm1:4.6 arm2:4.7 | 1 | 0 | 0 | 0 |
| 48 | Mahnaz Momenzadeh2022 | Iran | RCT | 55 | 2.8 2.8 | arm1:3 arm2:2.58 | 1 | 1 | 1 | 1 |
| 49 | Loveleen Maan2023 | India | RCT | 100 | 4.2 | NA | 1 | 0 | 1 | 0 |
| 50 | S D Maasdorp2021 | South Africa | retrospective study | 16 | NA | arm1:1.9 arm2:2 | 1 | 0 | 0 | 0 |
| 51 | Yaakov Dickstein2018 | Greece | RCT | 266 | NA | arm1:5 arm2:6 | 1 | 0 | 0 | 0 |
| 52 | Davide Fiore Bavaro2023 | Italy | retrospective study | 118 | NA | 10 | 1 | 1 | 0 | 1 |
| 53 | Lidia Dalfino2023 | Italy | RCT | 90 | 4.2 | 10 | 1 | 1 | 0 | 1 |
| 54 | A Russo 2023 | Italy | retrospective study | 73 | 4.2 | 5.9 | 1 | 0 | 0 | 0 |
| 55 | Maria Mazzitelli2023 | Italy | retrospective study | 111 | 4.2 | 7 | 1 | 1 | 1 | 1 |
| 56 | Wasan Katip2019 | Thailand | RCT | 255 | 4.2 4.2 | arm1:4.72 arm2:4.95 | 1 | 1 | 1 | 1 |
| 57 | Emine Alp2017 | Turkey | retrospective study | 52 | 4.2 4.2 | arm1:3.93 arm2:3.64 | 1 | 1 | 1 | 1 |
| 58 | 2Lidia Dalfino012 | Italy | RCT | 25 | 4 | 5.7 | 0 | 1 | 0 | 1 |
| 59 | Katip, W2024 | Thailand | retrospective study | 261 | 4.2 4.2 | arm1:5.18 arm2:5.39 | 1 | 1 | 1 | 1 |
| 60 | Katip, W 2022 | Thailand | retrospective study | 379 | 4.2 4.2 | arm1:5.69 arm2:5.29 | 1 | 1 | 1 | 1 |
| 61 | Junsu Choe2019 | Korea | retrospective study | 191 | 2.9 (2.1–4.3) 3.9 (2.9–5.0) 3.1 (2.2–4.1) | arm1:5.16 arm2:4.65 arm3:5 | 1 | 1 | 1 | 1 |
| 62 | Wasan Katip2017 | Thailand | retrospective study | 102 | 4.2(首剂加倍） 4.2 | arm1:6.1 arm2:7.44 | 0 | 1 | 1 | 1 |
| 63 | Ali S Omrani2015 | Saudi Arabia | RCT | 76 | 4.2(首剂加倍） | arm1:5.86 arm2:3.94 | 0 | 1 | 1 | 1 |
| 64 | Maria Helena Rigatto2016 | Brazil | RCT | 491 | 4.2 2.1 | arm1:2 arm2:4 | 0 | 0 | 0 | 1 |
| 65 | Ryan K Shields2017 | USA | retrospective study | 249 | NA | arm1:6 arm2:6 | 0 | 0 | 0 | 1 |
| 66 | Sunmi Jung2019 | Korea | retrospective study | 153 | 5.1 | 5 | 0 | 1 | 1 | 1 |
| 67 | Yong Kyun Kim2017 | Korea | retrospective study | 219 | 3.15-4.2 3.5(2.1-4.2) | arm1:5.35 arm2:5.25 | 1 | 1 | 1 | 1 |
| 68 | Jun-Yuan Zheng2020 | Taiwan | retrospective study | 183 | NA | 5.91 | 1 | 1 | 1 | 1 |
| 69 | Reinout Naesens2011 | Belgium | retrospective study | 26 | NA 2.9 2.9 | NA | 1 | 1 | 0 | 0 |
| 70 | Omid Moradi Moghaddam2019 | Iran | retrospective study | 114 | 4.14 4.1 | NA | 1 | 0 | 0 | 0 |
| 71 | I P Korbila 2010 | Greece | retrospective study | 121 | 6.3 5.1 | arm1:5.18 arm2:4.06 | 1 | 1 | 0 | 0 |
| 72 | M J Pérez-Pedrero2011 | Spain | retrospective study | 54 | NA | NA | 0 | 1 | 1 | 0 |
| 73 | Meltem Polat2015 | Turkey | retrospective study | 50 | 2.5-5   2.5-5 | arm1:1.13 arm2:1.16 | 1 | 1 | 1 | 1 |
| 74 | Mario Tumbarello2013 | Italy | retrospective study | 208 | NA | NA | 0 | 1 | 1 | 0 |

note: 0 = Unvailable

1= Available
