# Supplementary material for: Factors affecting the effectiveness and safety of colistin in treating drug-resistant gram-negative bacterial infections: a meta-analysis
Source: Front Pharmacol. 2025 Oct 29;16:1625595. doi: 10.3389/fphar.2025.1625595 (PMC12605452; doi:10.3389/fphar.2025.1625595)
Supplement: Supplementary file 1 [file DataSheet1.zip › Supplementary/Supplementary Material 5-Egger's test.docx]

1. **Egger’s Test of Overall Mortality VS Dose**
   1. Subgroup of high dose

- 1. Subgroup of low dose

1. **Egger’s Test of Overall Mortality VS ACCI**
   1. Subgroup of ACCI<5


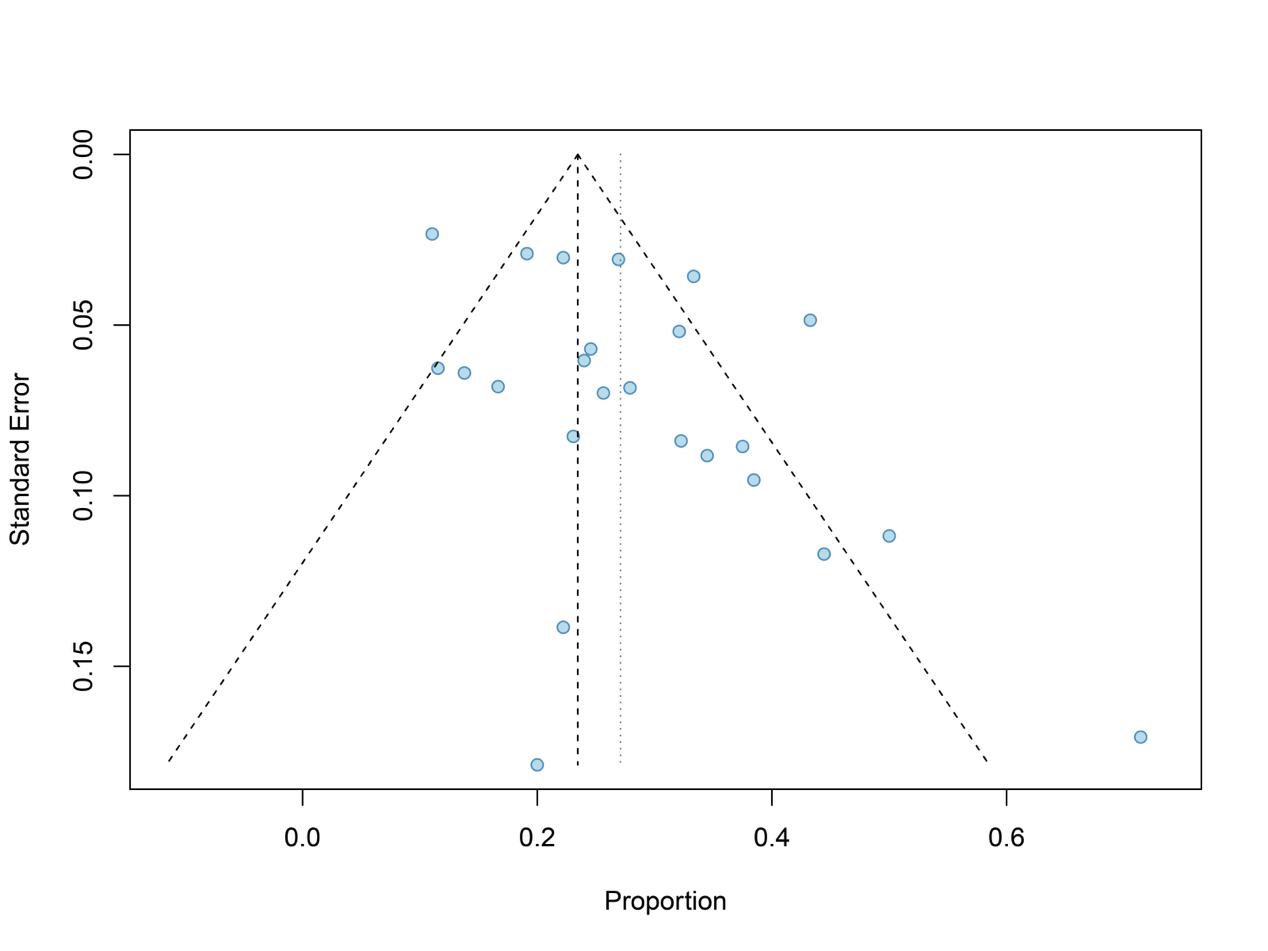


2.2Subgroup of ACCI=5~6


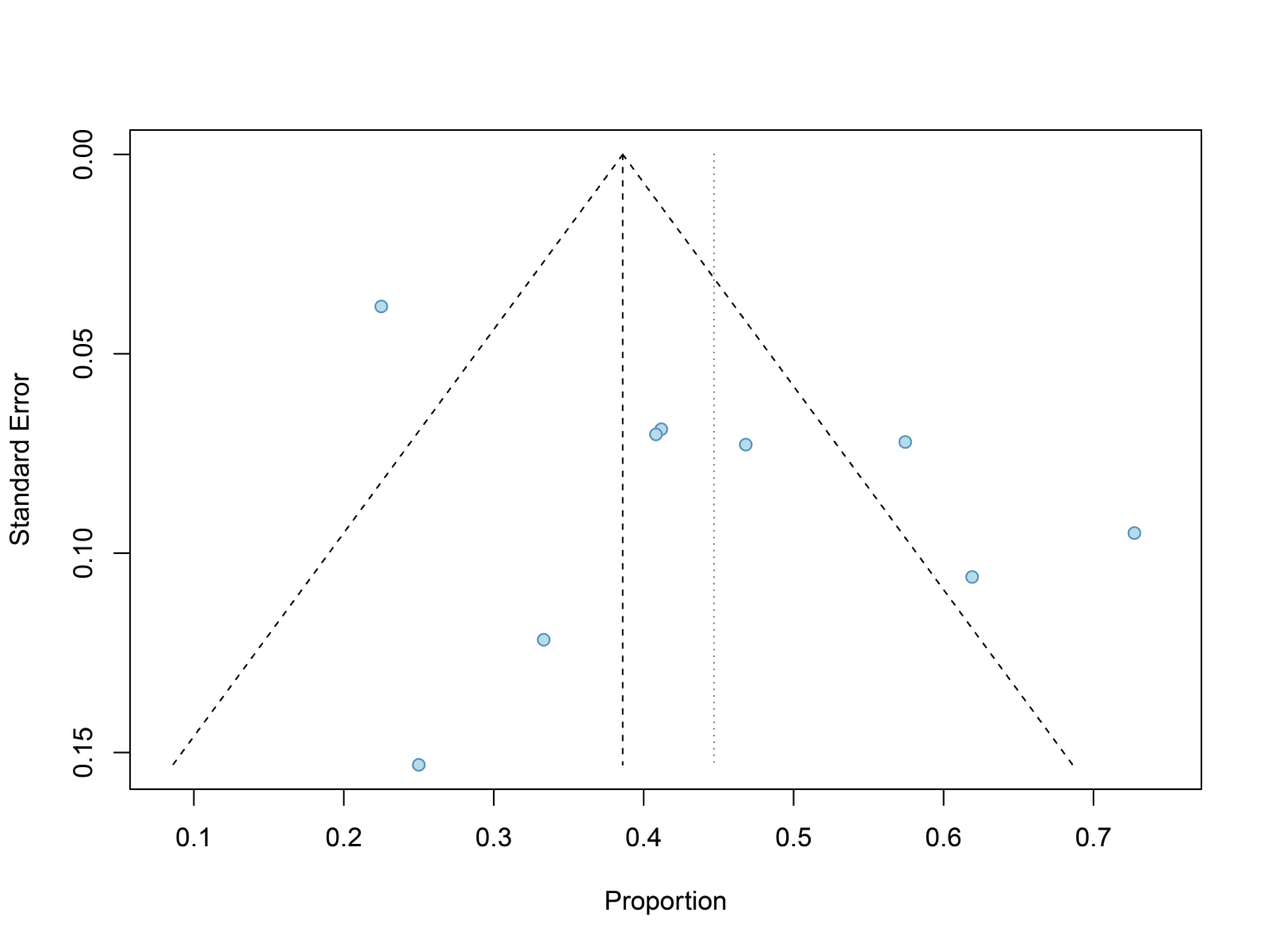


2.3Subgroup of ACCI>7


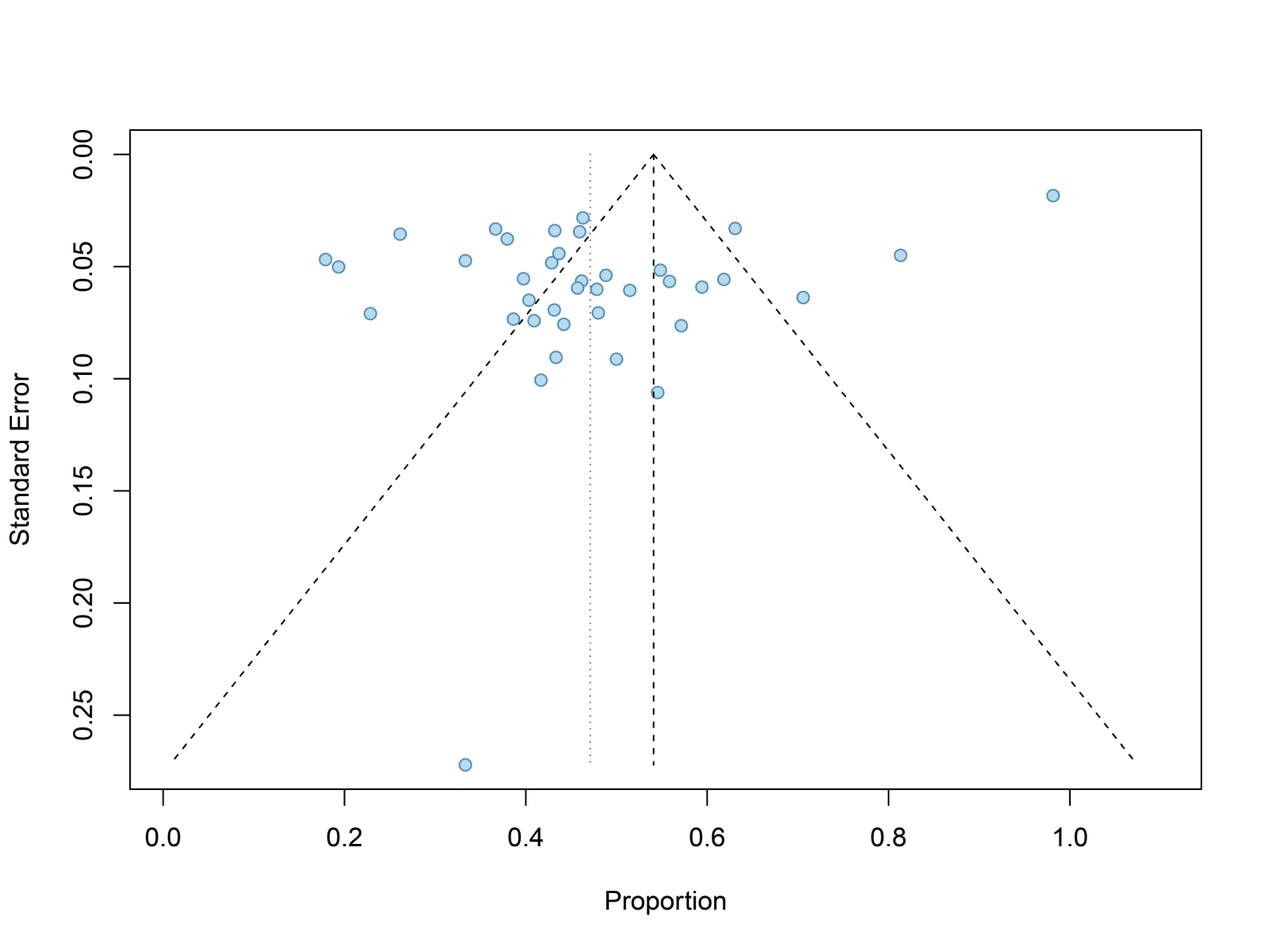


**3.Egger’s Test of Overall Mortality VS Antibiotic Drug co-therapy**

- 1. Monotherapy

- 1. Drug co-therapy --Mixed antibiotics

- 1. Drug co-therapy -- Carbapenems

- 1. Drug co-therapy -- Rifampicin

- 1. Drug co-therapy -- Tigecycline

- 1. Drug co-therapy -- Quinolones

**4.Egger’s Test of Overall Mortality VS Bacteria**

4.1Acinetobacter baumannii

4.2 Pseudomonas aeruginosa

4.3 Enterobacteriaceae

4.4 Klebsiella pneumoniae

4.5 Mixed bacteria

**5． Egger’s Test of Overall Mortality VS Mode of administration**

5.1 ivgtt

5.. ivgtt+ih

5.3 ih

**6.Egger’s Test of Overall Mortality VS dosage**

6.1 Loading dose

6.2 Maintenance dose
