# Supplementary material for: Factors affecting the effectiveness and safety of colistin in treating drug-resistant gram-negative bacterial infections: a meta-analysis
Source: Front Pharmacol. 2025 Oct 29;16:1625595. doi: 10.3389/fphar.2025.1625595 (PMC12605452; doi:10.3389/fphar.2025.1625595)
Supplement: Supplementary file 1 [file DataSheet1.zip › Supplementary/Supplementary Material 1- search strategy.docx]

**Table S1. Search strategy**

| **PUBMED** | | |
| --- | --- | --- |
| Search number | Query | Results |
| 32 | #30 not #31 | 354 |
| 31 | #28 not #29 | 2 |
| 30 | #28 not #29 | 356 |
| 29 | #6 and #21 and #27 | 54 |
| 28 | #6 and #21 and #27 | 410 |
| 27 | #22 or #23 or #24 or #25 or #26 | 3,008,007 |
| 26 | randomized controlled trial[Publication Type] | 630,320 |
| 25 | randomized controlled trial[MeSH Terms] | 181,297 |
| 24 | placebo | 282,148 |
| 23 | trial | 2,240,029 |
| 22 | random* | 1,836,715 |
| 21 | #7 or #8 or #9 or #10 or #11 or #12 or #13 or #14 or #15 or #16 or #17 or #18 or #19 or #20 | 205,469 |
| 20 | Multidrug resistance | 133,904 |
| 19 | extensively drug resistance | 90,183 |
| 18 | Carbapenem-resistant gram-negative bacteria | 7,796 |
| 17 | Carbapenem-resistant Enterobacteriaceae | 4,557 |
| 16 | Carbapenem-resistant Klebsiella pneumoniae | 4,289 |
| 15 | Carbapenem-resistant Acinetobacter baumannii | 2,689 |
| 14 | Carbapenem-resistant Pseudomonas aeruginosa | 1,939 |
| 13 | MDR | 38,339 |
| 12 | XDR | 4,117 |
| 11 | CRAB | 14,900 |
| 10 | CRPA | 479 |
| 9 | CRKP | 1,248 |
| 8 | CRE | 30,379 |
| 7 | CRO | 6,932 |
| 6 | #1 or #2 or #3 or #4 or #5 | 42,271 |
| 5 | CMS | 30,313 |
| 4 | Colistimethate sodium | 315 |
| 3 | polymyxin E sodium methanesulfonate | 38 |
| 2 | Colistin | 12,140 |
| 1 | Colistin[MeSH Terms] | 6,142 |

| **science of web** | | |
| --- | --- | --- |
| 1 | TS=(Colistin or polymyxin E sodium methanesulfonate or Colistimethate sodium or CMS) and Preprint Citation Index (Exclude – Database) | 58132 |
| 2 | TS=(CRO OR CRE OR CRPA OR CRKP OR CRPA OR CRAB OR XDR OR MDR) and Preprint Citation Index (Exclude – Database) | 163825 |
| 3 | TS=(Carbapenem-resistant gram-negative bacteria OR Carbapenem-resistant Enterobacteriaceae OR Carbapenem-resistant Klebsiella pneumoniae OR Carbapenem-resistant Acinetobacter baumannii OR Carbapenem-resistant Pseudomonas aeruginosa OR extensively drug resistance OR Multidrug resistance) and Preprint Citation Index (Exclude – Database) | 132793 |
| 4 | #2 OR #3 and Preprint Citation Index (Exclude – Database) | 260814 |
| 5 | TS=(randomized controlled trial) and Preprint Citation Index (Exclude – Database) | 801229 |
| 6 | #1 AND #4 AND #5 and Preprint Citation Index (Exclude – Database) | 186 |
| 7 | #1 AND #4 AND #5 and Preprint Citation Index (Exclude – Database) and Korean or Turkish (Exclude – Languages) | 184 |

| **cochrane library** | | |
| --- | --- | --- |
| #1 | MeSH descriptor: [Colistin] explode all trees | 239 |
| #2 | (colistin ):ti,ab,kw OR (polymyxin E sodium methanesulfonate):ti,ab,kw OR (CMS):ti,ab,kw OR (Colistimethate sodium):ti,ab,kw | 2530 |
| #3 | (CRO ):ti,ab,kw OR (CRE):ti,ab,kw OR (CRPA):ti,ab,kw OR (CRAB):ti,ab,kw OR (CRKP):ti,ab,kw OR (XDR):ti,ab,kw OR (MDR):ti,ab,kw | 2455 |
| #4 | (Carbapenem-resistant Pseudomonas aeruginosa ):ti,ab,kw OR (Carbapenem-resistant Acinetobacter baumannii):ti,ab,kw OR (Carbapenem-resistant Klebsiella pneumoniae):ti,ab,kw OR (Carbapenem-resistant Enterobacteriaceae):ti,ab,kw OR (Carbapenem-resistant gram-negative bacteria ):ti,ab,kw OR (extensively drug resistance):ti,ab,kw OR (Multidrug resistance):ti,ab,kw | 1656 |
| #5 | #1 OR #2 | 2530 |
| #6 | #3 OR #4 | 3652 |
| #7 | #5 AND #6 | 179 |

| Embase | | |
| --- | --- | --- |
| #9 | #7 AND #8 | 124 |
| #8 | 'randomized controlled trial (topic)'/exp OR 'randomized controlled trial (topic)' | 286508 |
| #7 | #3 AND #6 | 9857 |
| #6 | #4 OR #5 | 216960 |
| #5 | 'carbapenem-resistant gram-negative bacteria' OR 'carbapenem-resistant enterobacteriaceae'/exp OR 'carbapenem-resistant enterobacteriaceae' OR 'carbapenem-resistant klebsiella pneumoniae'/exp OR 'carbapenem-resistant klebsiella pneumoniae' OR 'carbapenem-resistant acinetobacter baumannii'/exp OR 'carbapenem-resistant acinetobacter baumannii' OR 'carbapenem-resistant pseudomonas aeruginosa'/exp OR 'carbapenem-resistant pseudomonas aeruginosa' OR 'extensively drug resistance'/exp OR 'extensively drug resistance' OR 'multidrug resistance'/exp OR 'multidrug resistance' | 112335 |
| #4 | 'cro' OR 'cre'/exp OR 'cre' OR 'crkp' OR 'crpa' OR 'crab'/exp OR 'crab' OR 'xdr' OR 'mdr' | 133199 |
| #3 | #1 OR #2 | 86255 |
| #2 | 'colistin'/exp OR 'colistin' | 31462 |
| #1 | 'colistin'/exp OR 'colistin' OR 'polymyxin e sodium methanesulfonate' OR 'colistimethate sodium'/exp OR 'colistimethate sodium' OR 'cms' | 86255 |
