# Supplementary material for: Factors affecting the effectiveness and safety of colistin in treating drug-resistant gram-negative bacterial infections: a meta-analysis
Source: Front Pharmacol. 2025 Oct 29;16:1625595. doi: 10.3389/fphar.2025.1625595 (PMC12605452; doi:10.3389/fphar.2025.1625595)
Supplement: Supplementary file 1 [file DataSheet1.zip › Supplementary/Supplementary Material 6- sensitivity analyses.docx]

**1.Dose（high-dose vs low-dose）**

**2.ACCI**


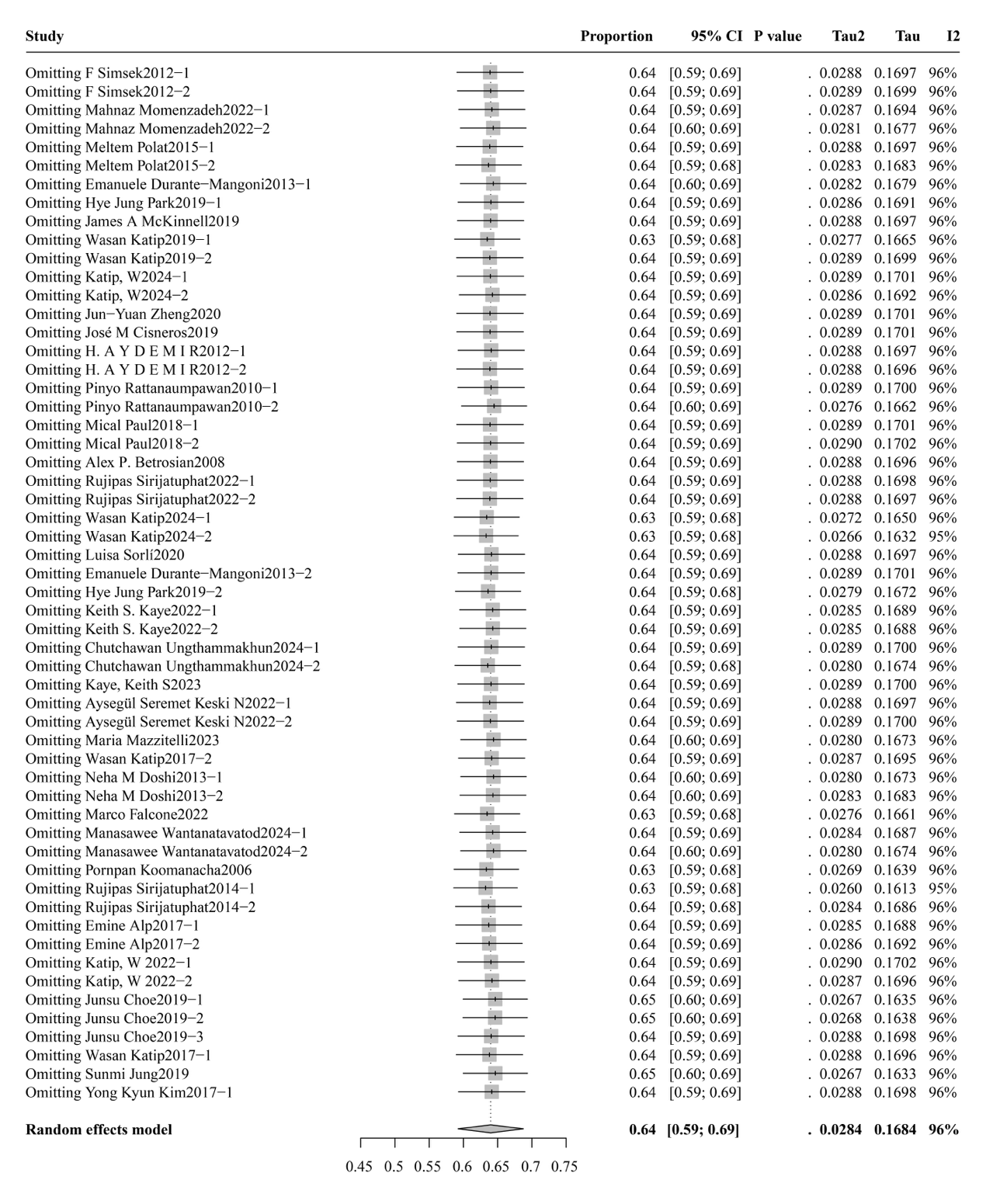


**3. Antibiotic Drug co-therapy**

**4. Bacteria**

**5. Mode of administration**

**6. Dosage（a loading dose）**
