# Supplementary material for: Factors affecting the effectiveness and safety of colistin in treating drug-resistant gram-negative bacterial infections: a meta-analysis
Source: Front Pharmacol. 2025 Oct 29;16:1625595. doi: 10.3389/fphar.2025.1625595 (PMC12605452; doi:10.3389/fphar.2025.1625595)

**Table S2. Quality evaluation of the eligible studies with Newcastle-Ottawa scale(Observational studies).**

|  | Selection | | | | Compar abilit | outcome | | | score |
| --- | --- | --- | --- | --- | --- | --- | --- | --- | --- |
| Study | Representa tiveness | Selec tion of non- expo sed | Ascertai nment of exposure | Outc ome not prese nt at start | Compar ability on most importa nt factors or other risk factor | Assess ment of outcome | Lon geno ugh foll ow- up | Adequacy (complet eness)of follow-up |  |
| 1^[1]^ | 1 | 1 | 1 | 1 | 0 | 1 | 1 | 1 | 7 |
| 2^[2]^ | 1 | 1 | 1 | 1 | 2 | 1 | 1 | 1 | 9 |
| 3^[3]^ | 1 | 1 | 1 | 1 | 2 | 1 | 1 | 1 | 9 |
| 4^[4]^ | 1 | 1 | 1 | 1 | 2 | 1 | 1 | 1 | 9 |
| 5^[5]^ | 1 | 1 | 1 | 1 | 2 | 1 | 1 | 1 | 9 |
| 6^[6]^ | 1 | 1 | 1 | 1 | 1 | 1 | 1 | 1 | 8 |
| 7^[7]^ | 1 | 1 | 1 | 1 | 1 | 1 | 1 | 0 | 7 |
| 8^[8]^ | 1 | 1 | 1 | 1 | 1 | 1 | 1 | 0 | 7 |
| 9^[9]^ | 1 | 1 | 1 | 1 | 1 | 1 | 1 | 1 | 8 |
| 10^[10]^ | 1 | 1 | 1 | 1 | 1 | 1 | 1 | 1 | 8 |
| 11^[11]^ | 1 | 1 | 1 | 1 | 1 | 1 | 1 | 0 | 7 |
| 12^[12]^ | 1 | 1 | 1 | 1 | 2 | 1 | 1 | 1 | 9 |
| 13^[13]^ | 1 | 1 | 1 | 1 | 1 | 1 | 1 | 1 | 8 |
| 14^[14]^ | 1 | 1 | 1 | 1 | 1 | 1 | 1 | 1 | 8 |
| 15^[15]^ | 1 | 1 | 1 | 1 | 2 | 1 | 1 | 1 | 9 |
| 16^[16]^ | 1 | 1 | 1 | 1 | 2 | 1 | 1 | 1 | 9 |
| 17^[17]^ | 1 | 1 | 1 | 1 | 1 | 1 | 1 | 0 | 7 |
| 18^[18]^ | 1 | 1 | 1 | 1 | 1 | 1 | 1 | 1 | 8 |
| 19^[19]^ | 1 | 1 | 1 | 1 | 2 | 1 | 1 | 1 | 9 |
| 20^[20]^ | 1 | 1 | 1 | 1 | 0 | 1 | 1 | 1 | 7 |
| 21^[21]^ | 1 | 1 | 1 | 1 | 1 | 1 | 1 | 1 | 8 |
| 22^[22]^ | 1 | 1 | 1 | 1 | 1 | 1 | 1 | 1 | 8 |
| 23^[23]^ | 1 | 1 | 1 | 1 | 1 | 1 | 1 | 1 | 8 |
| 24^[24]^ | 1 | 1 | 1 | 1 | 1 | 1 | 1 | 0 | 7 |
| 25^[25]^ | 1 | 1 | 1 | 1 | 1 | 1 | 1 | 1 | 8 |
| 26^[26]^ | 1 | 1 | 1 | 1 | 2 | 1 | 0 | 0 | 7 |
| 27^[27]^ | 1 | 1 | 1 | 1 | 1 | 1 | 1 | 1 | 7 |
| 28^[28]^ | 1 | 1 | 1 | 1 | 1 | 1 | 1 | 1 | 8 |
| 29^[29]^ | 1 | 1 | 1 | 1 | 2 | 1 | 1 | 1 | 9 |
| 30^[30]^ | 1 | 1 | 1 | 1 | 0 | 1 | 1 | 1 | 7 |
| 31^[31]^ | 1 | 1 | 1 | 1 | 2 | 1 | 1 | 1 | 9 |
| 32^[32]^ | 1 | 1 | 1 | 1 | 1 | 1 | 1 | 1 | 8 |
| 33^[33]^ | 1 | 1 | 1 | 1 | 2 | 1 | 1 | 1 | 9 |
| 34^[34]^ | 1 | 1 | 1 | 1 | 1 | 1 | 1 | 0 | 7 |
| 35^[35]^ | 1 | 1 | 1 | 1 | 0 | 1 | 1 | 1 | 7 |
| 36^[36]^ | 1 | 1 | 1 | 1 | 0 | 1 | 1 | 1 | 7 |
| 37^[37]^ | 1 | 1 | 1 | 1 | 2 | 1 | 1 | 1 | 9 |
| 38^[38]^ | 1 | 1 | 1 | 1 | 1 | 1 | 1 | 1 | 8 |
| 39^[39]^ | 1 | 1 | 1 | 1 | 1 | 1 | 1 | 1 | 8 |
| 40^[40]^ | 1 | 1 | 1 | 1 | 2 | 1 | 1 | 1 | 9 |
| 41^[41]^ | 1 | 1 | 1 | 1 | 0 | 1 | 1 | 1 | 7 |

[1] Wacharachaisurapol N, Kawichai S, Chanakul A, et al. No increased acute kidney injury rate through giving an intravenous colistin loading dose in pediatric patients[J]. International journal of infectious diseases: IJID: official publication of the International Society for Infectious Diseases, 2021, 106: 91-97.

[2] Katip W, Rayanakorn A, Oberdorfer P, et al. Comparative effectiveness and mortality of colistin monotherapy versus colistin-fosfomycin combination therapy for the treatment of carbapenem-resistant enterobacteriaceae (CRE) infections: A propensity score analysis[J]. Journal of Infection and Public Health, 2024, 17(5): 727-734.

[3] Sorlí L, Luque S, Li J, et al. Colistin for the treatment of urinary tract infections caused by extremely drug-resistant pseudomonas aeruginosa: Dose is critical[J]. Journal of Infection, 2019, 79(3): 253-261.

[4] Alexander C. A retrospective case-control study of eravacycline for the treatment of carbapenem-resistant acinetobacter infections in patients with burn injuries[J]. Journal of Burn Care & Research: Official Publication of the American Burn Association, 2023, 45(2): 487.

[5] Durante-Mangoni E, Andini R, Signoriello S, et al. Acute kidney injury during colistin therapy: A prospective study in patients with extensively-drug resistant acinetobacter baumannii infections[J]. Clinical Microbiology and Infection, 2016, 22(12): 984-989.

[6] Doshi N M, Cook C H, Mount K L, et al. Adjunctive aerosolized colistin for multi-drug resistant gram-negative pneumonia in the critically ill: A retrospective study[J]. BMC anesthesiology, 2013, 13(1): 45.

[7] Falcone M, Tiseo G, Leonildi A, et al. Cefiderocol- compared to colistin-based regimens for the treatment of severe infections caused by carbapenem-resistant acinetobacter baumannii[J]. Antimicrobial Agents and Chemotherapy, 2022, 66(5): e0214221.

[8] Tascini C, Gemignani G, Palumbo F, et al. Clinical and microbiological efficacy of colistin therapy alone or in combination as treatment for multidrug resistant pseudomonas aeruginosa diabetic foot infections with or without osteomyelitis[J]. Journal of Chemotherapy (Florence, Italy), 2006, 18(6): 648-651.

[9] Simsek F, Gedik H, Yildirmak M T, et al. Colistin against colistin-only-susceptible acinetobacter baumannii-related infections: Monotherapy or combination therapy?[J]. Indian Journal of Medical Microbiology, 2012, 30(4): 448-452.

[10] Motaouakkil S, Charra B, Hachimi A, et al. Colistin and rifampicin in the treatment of nosocomial infections from multiresistant acinetobacter baumannii[J]. Journal of Infection, 2006, 53(4): 274-278.

[11] Álvarez-Marín R, López-Rojas R, Márquez J A, et al. Colistin dosage without loading dose is efficacious when treating carbapenem-resistant acinetobacter baumannii ventilator-associated pneumonia caused by strains with high susceptibility to colistin[J]. PLOS One, 2016, 11(12): e0168468.

[12] van Duin D, Lok J J, Earley M, et al. Colistin versus ceftazidime-avibactam in the treatment of infections due to carbapenem-resistant enterobacteriaceae[J]. Clinical Infectious Diseases: An Official Publication of the Infectious Diseases Society of America, 2018, 66(2): 163-171.

[13] Kasiakou S K, Michalopoulos A, Soteriades E S, et al. Combination therapy with intravenous colistin for management of infections due to multidrug-resistant gram-negative bacteria in patients without cystic fibrosis[J]. Antimicrobial Agents and Chemotherapy, 2005, 49(8): 3136.

[14] Durakovic N, Radojcic V, Boban A, et al. Efficacy and safety of colistin in the treatment of infections caused by multidrug-resistant pseudomonas aeruginosa in patients with hematologic malignancy: A matched pair analysis[J]. Internal Medicine (Tokyo, Japan), 2011, 50(9): 1009-1013.

[15] Katip W, Meechoui M, Thawornwittayakom P, et al. Efficacy and safety of high loading dose of colistin in multidrug-resistant *acinetobacter baumannii* : a prospective cohort study[J]. Journal of Intensive Care Medicine, 2019, 34(11-12): 996-1002.

[16] Russo A, Bassetti M, Bellelli V, et al. Efficacy of a fosfomycin-containing regimen for treatment of severe pneumonia caused by multidrug-resistant acinetobacter baumannii: A prospective, observational study[J]. Infectious Diseases and Therapy, 2020, 10(1): 187.

[17] Kapoor K, Jajoo M, Dublish S, et al. Intravenous colistin for multidrug-resistant gram-negative infections in critically ill pediatric patients[J]. Pediatric Critical Care Medicine: A Journal of the Society of Critical Care Medicine and the World Federation of Pediatric Intensive and Critical Care Societies, 2013, 14(6): e268-272.

[18] Markou N, Apostolakos H, Koumoudiou C, et al. Intravenous colistin in the treatment of sepsis from multiresistant gram-negative bacilli in critically ill patients[J]. Critical Care (London, England), 2003, 7(5): R78-83.

[19] Keski N A S, Seyman D, Önder K D, et al. Investigation of effect of the colistin loading dosage on the clinical, microbiological, and laboratory results in acinetobacter baumannii ventilator-associated pneumonia /pneumonia[J]. International Journal of Clinical Practice, 2022, 2022: 5437850.

[20] Ghafur A, Devarajan V, Raja T, et al. Monotherapy versus combination therapy against nonbacteremic carbapenem-resistant gram-negative infections: A retrospective observational study[J]. Indian Journal of Critical Care Medicine: Peer-Reviewed, Official Publication of Indian Society of Critical Care Medicine, 2017, 21(12): 825-829.

[21] Amat T, Gutiérrez-Pizarraya A, Machuca I, et al. The combined use of tigecycline with high-dose colistin might not be associated with higher survival in critically ill patients with bacteraemia due to carbapenem-resistant acinetobacter baumannii[J]. Clinical Microbiology and Infection, 2018, 24(6): 630-634.

[22] Maasdorp S D, Potgieter S, Glover E, et al. Treatment outcomes of acinetobacter baumannii -associated pneumonia and/or bacteraemia at the intensive care unit of universitas academic hospital, bloemfontein, south africa[J]. African Journal of Thoracic and Critical Care Medicine, 2021, 27(1).

[23] Bavaro D F, Papagni R, Belati A, et al. Cefiderocol versus colistin for the treatment of carbapenem-resistant acinetobacter baumannii complex bloodstream infections: A retrospective, propensity-score adjusted, monocentric cohort study[J]. Infectious Diseases and Therapy, 2023, 12(8): 2147.

[24] Russo A, Bruni A, Gullì S, et al. Efficacy of cefiderocol- vs colistin-containing regimen for treatment of bacteraemic ventilator-associated pneumonia caused by carbapenem-resistant acinetobacter baumannii in patients with COVID-19[J]. International Journal of Antimicrobial Agents, 2023, 62(1): 106825.

[25] Mazzitelli M, Gregori D, Sasset L, et al. Cefiderocol-based versus colistin-based regimens for severe carbapenem-resistant acinetobacter baumannii infections: A propensity score-weighted, retrospective cohort study during the first two years of the COVID-19 pandemic[J]. Microorganisms, 2023, 11(4): 984.

[26] Alp E, Eren E, Elay G, et al. Efficacy of loading dose of colistin in acinetobacter baumannii ventilator-associated pneumonia[J]. Le Infezioni in Medicina, 2017, 25(4): 311-319.

[27] Katip W, Rayanakorn A, Sornsuvit C, et al. High-loading-dose colistin with nebulized administration for carbapenem-resistant acinetobacter baumannii pneumonia in critically ill patients: A retrospective cohort study[J]. Antibiotics, 2024, 13(3): 287.

[28] Katip W, Oberdorfer P, Kasatpibal N. Effectiveness and nephrotoxicity of loading dose colistin–meropenem versus loading dose colistin–imipenem in the treatment of carbapenem-resistant acinetobacter baumannii infection[J]. Pharmaceutics, 2022, 14(6): 1266.

[29] Choe J, Sohn Y M, Jeong S H, et al. Inhalation with intravenous loading dose of colistin in critically ill patients with pneumonia caused by carbapenem-resistant gram-negative bacteria[J]. Therapeutic Advances in Respiratory Disease, 2019, 13: 1753466619885529.

[30] Katip W, Uitrakul S, Oberdorfer P. Clinical outcomes and nephrotoxicity of colistin loading dose for treatment of extensively drug-resistant acinetobacter baumannii in cancer patients[J]. Infection and Drug Resistance, 2017, 10: 293.

[31] Shields R K, Anand R, Clarke L G, et al. Defining the incidence and risk factors of colistin-induced acute kidney injury by KDIGO criteria[J]. PLoS ONE, 2017, 12(3): e0173286.

[32] Jung S, Chung E K, Jun M S, et al. Differences in colistin administration and bacterial and treatment outcomes in critically ill patients[J]. Scientific Reports, 2019, 9: 8781.

[33] Kim Y K, Lee J H, Lee H K, et al. Efficacy of nebulized colistin-based therapy without concurrent intravenous colistin for ventilator-associated pneumonia caused by carbapenem-resistant acinetobacter baumannii[J]. Journal of Thoracic Disease, 2017, 9(3): 555.

[34] Zheng J Y, Huang S S, Huang S H, et al. Colistin for pneumonia involving multidrug-resistant acinetobacter calcoaceticus-acinetobacter baumannii complex[J]. Journal of Microbiology, Immunology, and Infection = Wei Mian Yu Gan Ran Za Zhi, 2020, 53(6): 854-865.

[35] Naesens R, Vlieghe E, Verbrugghe W, et al. A retrospective observational study on the efficacy of colistin by inhalation as compared to parenteral administration for the treatment of nosocomial pneumonia associated with multidrug-resistant pseudomonas aeruginosa[J]. BMC Infectious Diseases, 2011, 11: 317.

[36] Moradi Moghaddam O, Niakan Lahiji M, Talebi-Taher M, et al. Effect of inhaled colistin on the treatment of ventilator-associated pneumonia due to multi-drug resistant acinetobacter[J]. Tanaffos, 2019, 18(1): 66-73.

[37] Korbila I P, Michalopoulos A, Rafailidis P I, et al. Inhaled colistin as adjunctive therapy to intravenous colistin for the treatment of microbiologically documented ventilator-associated pneumonia: A comparative cohort study[J]. Clinical Microbiology and Infection, 2010, 16(8): 1230-1236.

[38] Karvouniaris M, Makris D, Zygoulis P, et al. Nebulised colistin for ventilator-associated pneumonia prevention[J]. European Respiratory Journal, 2015, 46(6): 1732-1739.

[39] Polat M, Kara S S, Tapısız A, et al. Treatment of ventilator-associated pneumonia using intravenous colistin alone or in combination with inhaled colistin in critically ill children[J]. Paediatric Drugs, 2015, 17(4): 323-330.

[40] Tumbarello M, De Pascale G, Trecarichi E M, et al. Effect of aerosolized colistin as adjunctive treatment on the outcomes of microbiologically documented ventilator-associated pneumonia caused by colistin-only susceptible gram-negative bacteria[J]. Chest, 2013, 144(6): 1768-1775.

[41] Levin A S, Barone A A, Penço J, et al. Intravenous colistin as therapy for nosocomial infections caused by multidrug-resistant pseudomonas aeruginosa and acinetobacter baumannii[J]. Clinical Infectious Diseases: An Official Publication of the Infectious Diseases Society of America, 1999, 28(5): 1008-1011.

**picture1：Risk of bias graph(RCT）**


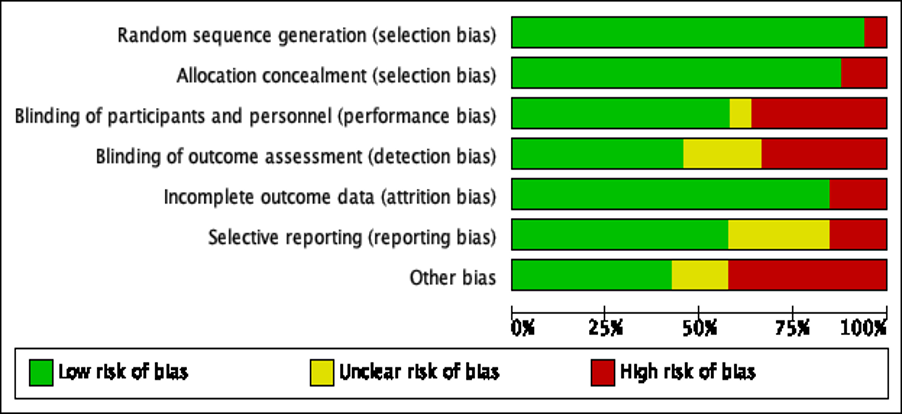


**Picture2：Risk of bias summary(RCT)**


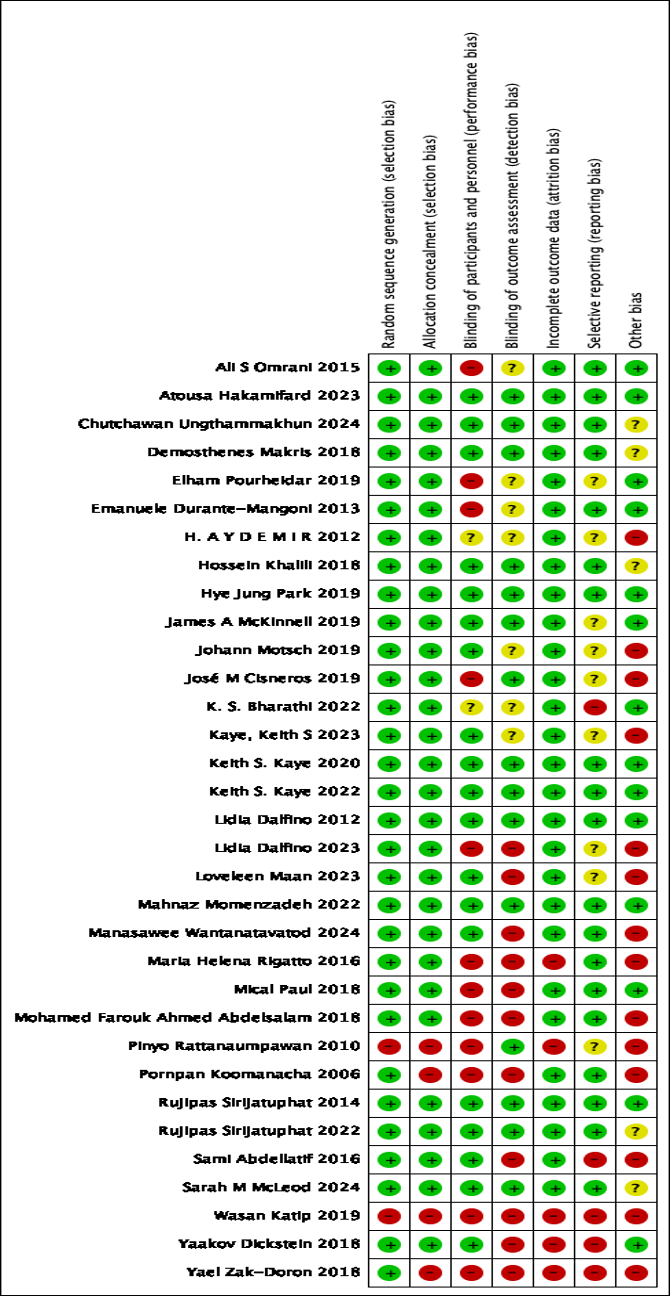

Supplement: Supplementary file 1 [file DataSheet1.zip › Supplementary/Supplementary Material 4-The risk of bias.docx]
